# Effects of Remote Web-Based Interventions on the Physiological and Psychological States of Patients With Cancer: Systematic Review With Meta-Analysis

**DOI:** 10.2196/71196

**Published:** 2025-06-12

**Authors:** Lv Tian, Yixuan Wen, Jingmiao Li, Jiexin Guan, Tao Li, Jun Fan

**Affiliations:** 1 Institute of Fundamental and Frontier Sciences University of Electronic Science and Technology of China Chengdu China; 2 Shimmer Center Tianfu Jiangxi Laboratory Chengdu China

**Keywords:** meta-analysis, cancer patients, pain, depression, anxiety, quality of life, remote web-based intervention, PRISMA

## Abstract

**Background:**

Patients with cancer may experience physiological and psychological adverse reactions, such as fatigue, pain, anxiety, and depression, which seriously affect their quality of life. Research has shown that remote interventions based on apps or miniprograms may help improve the physiological and mental health of patients with cancer. However, due to the limited number of relevant studies, the impact of web-based interventions in cancer management remains unclear.

**Objective:**

We aimed to determine the efficacy of interventions based on apps, miniprograms, or other web-based tools on the physiological (body pain and fatigue) and psychological (anxiety and depression) states and the quality of life of patients with cancer.

**Methods:**

We conducted electronic literature searches in PubMed, Scopus, Web of Science, the Cochrane Library, CINAHL, and EMBASE databases. The search period spanned from the inception of each database to October 15, 2024. Two researchers independently conducted literature retrieval and data extraction. The risk of bias was assessed with the Cochrane risk-of-bias tool, and the quality of evidence was assessed according to the Grading of Recommendations Assessment, Development, and Evaluation (GRADE). All statistical analyses were performed using Review Manager version 5.4.

**Results:**

A total of 36 randomized controlled trials were included. The remote web-based interventions significantly improved the pain intensity (n=14, 39% studies; standardized mean difference [SMD] –0.39, 95% CI –0.64 to –0.14; *I*^2^=82%; GRADE rating=low) and fatigue status (n=11, 31% studies; SMD –0.52, 95% CI –0.95 to –0.09; *I*^2^=95%; GRADE rating=low) in patients with cancer. Regarding psychology, the results indicated that the remote web-based interventions significantly improved the anxiety (n=14, 39% studies; SMD –0.60, 95% CI –0.90 to –0.30; *I*^2^=91%; GRADE rating=low) and depressive state (n=10, 28% studies; SMD –0.36, 95% CI –0.58 to –0.14; *I*^2^=81%; GRADE rating=low) of patients with cancer. For quality of life, the results showed that the remote web-based interventions had a significant positive impact on the quality of life of patients with cancer (n=26, 72% studies; SMD 0.63, 95% CI 0.39-0.87; *I*^2^=92%; GRADE rating=low).

**Conclusions:**

The remote web-based interventions were effective in reducing the intensity of physiological pain, relieving fatigue, improving depression and anxiety, and improving the quality of life of patients with cancer. However, due to the low certainty of evidence, more rigorous randomized controlled trials are needed to validate these findings further.

**Trial Registration:**

PROSPERO CRD42024611768; https://www.crd.york.ac.uk/PROSPERO/view/CRD42024611768

## Introduction

Cancer is a highly prevalent disease globally. It is estimated that by 2025, more than 20 million people will be affected by cancer [1,2]. Cancer also poses a severe global health challenge, subjecting patients to immense physiological and psychological stress [3-5]. Although there are various treatment methods, such as surgery, radiotherapy, chemotherapy, targeted therapy, and immunotherapy, cancer remains an incurable disease that requires continuous care [6]. Unfortunately, these treatments are often accompanied by side effects, such as pain, fatigue, and sleep disorders [7]. There is a close and complex inherent relationship between the physiological condition and psychological state of patients with cancer [8-12]. Physiologically, the discomfort symptoms experienced by patients, such as pain, fatigue, and nausea, can trigger a chain reaction psychologically, leading to the emergence of negative emotions like anxiety and depression, as well as a significant increase in stress levels, thus seriously affecting the patient’s psychological states. At the same time, psychological distress and negative emotions can further exacerbate physiological discomfort symptoms, impede treatment compliance, and consequently have a negative impact on the overall quality of life of patients. The traditional model for managing side effects in patients with cancer mainly relies on face-to-face medical consultations, but this model may lack comprehensiveness or reliability [13]. For example, face-to-face medical consultations are restricted by geographic locations and medical resources. Patients in remote areas find it difficult to obtain timely and high-quality medical consultation services, and adverse reactions cannot be effectively managed. Time constraints make it challenging for physicians to comprehensively collect information on patients’ adverse reactions in their daily lives. Meanwhile, patients may forget some symptoms due to nervousness, which affect their comprehensive assessment of their conditions.

With the rapid development of digital information technology, many health management miniprograms that can be directly used on supporting platforms, such as WeChat and Alipay, have been developed. These miniprograms rely on the app software ecosystem and are characterized by lightweight design and convenience. Therefore, remote web-based interventions based on apps or miniprograms have become a promising treatment solution [2]. Remote web-based intervention is a medical support model based on digital technology, enabling symptom management through specialized applications, such as health management apps for patients with cancer, medical miniprograms, and web-based platforms such as professional medical websites. The related applications for remote web-based interventions have become promising tools for supporting patients’ self-management and improving health outcomes due to their acceptability, convenience, accessibility, personalization, and cost control [14]. This intervention model breaks through the spatiotemporal limitations of traditional health care, providing comprehensive services for patients with cancer, including health education, psychological support, symptom monitoring, and rehabilitation guidance. For example, research has shown that mobile health (mHealth) interventions can improve medication adherence, self-management participation, and health outcomes [15,16]. Through remote web-based interventions, patients can obtain various forms of support at home and receive medical services more cost-effectively [17,18].

In recent years, the management of side effects in patients with cancer through remote web-based interventions has received increasing attention. However, in current research, the efficacy of remote web-based interventions on the adverse reactions of patients with cancer is inconsistent. Regarding the management of physiological pain in patients with cancer, some studies have shown that remote web-based interventions can improve the intensity of physiological pain [19-22], yet other studies have pointed out that remote web-based interventions did not significantly improve the physiological pain of patients with cancer [23-25]. Some even indicated that remote web-based interventions could exacerbate the physiological pain of patients with cancer [26]. Regarding improving the psychological state of patients with cancer, some studies suggest that remote web-based interventions can improve the psychological state of patients with cancer [27,28], while others draw different conclusions [29-31]. In addition, remote web-based interventions have different impacts on the quality of life of patients with cancer [20,28,32,33]. However, research on remote web-based interventions in the physiological and mental management of patients with cancer remains limited. There is a lack of comprehensive and systematic reviews to reflect the impact of remote web-based interventions on patients with cancer. Therefore, based on these conflicting research results, we conducted a meta-analysis to clarify the clinical efficacy of remote web-based interventions on the physiological (pain and fatigue) and psychological (anxiety and depression) states and the quality of life of patients with cancer.

## Methods

### Study Design

This systematic review and meta-analysis was registered in PROSPERO (CRD42024611768), and the study was reported following the PRISMA (Preferred Reporting Items for Systematic Reviews and Meta-Analyses) guidelines (Multimedia Appendix 1) [34].

### Search Strategy

We conducted electronic literature searches in PubMed, Scopus, Web of Science, Cochrane Library, CINAHL, and EMBASE from the inception of each database to October 15, 2024. The search terms consisted of subject headings and free terms. The full search strategy and other relevant information are provided in Table S1 in Multimedia Appendix 2. Meanwhile, manual searches were carried out according to reference lists of relevant articles.

### Eligibility Criteria

Literature searches were limited to published English-language articles. Eligible studies had to meet the inclusion criteria provided in Textbox 1.

Studies meeting the inclusion criteria provided in Textbox 1 were included in this review.

**Inclusion criteria.**

- Population: the target population had to be patients with cancer, regardless of the type of cancer.
- Intervention: the intervention group adopted remote web-based interventions based on apps, miniprograms, or other web-based platforms.
- Comparison: the intervention in the control group was routine care or other non–web-based interventions.
- Outcomes: at least one outcome indicator had to be included—pain, anxiety, depression, fatigue, and quality of life (mean and SD).
- Study design: the type of studies included had to be randomized controlled trials.

### Study Selection

Search results from all databases were imported into EndNote X9 (Clarivate) software to remove duplicate studies and manage the research. Two independent reviewers (LT and YW) screened the titles and abstracts of the studies according to the inclusion criteria. After identifying potentially eligible studies, the full texts were screened again to determine the final included studies. Then, the 2 reviewers (LT and YW) discussed and reached a consensus. A third reviewer (JF) was available to assist and mediate throughout the process.

### Data Extraction

Data extraction followed the PRISMA statement to ensure a systematic approach. Two reviewers (LT and YW) independently extracted data using a pretested data extraction form, and a third author (JF) cross-checked the accuracy of the results. The data extracted included publication information (author, research year, and country); study design; characteristics of the study participants (sample size, age, sex, and type of cancer); duration of intervention; intervention methods in the intervention group and the control group, respectively; primary outcomes and assessment scales (methods); and main results. The primary outcome data were extracted as mean and SDs.

### Quality Assessment and Certainty of Evidence

Two authors (LT and YW) independently evaluated the risk of bias, methodological quality, and certainty of evidence of the included studies. In case of inconsistencies, discussions were held with a third reviewer (JF) until a consensus was reached. The Revised Cochrane Risk-of-Bias Tool was used to assess the risk of bias from the following aspects: randomization process, deviation from the intended interventions, missing outcome data, outcome measurement, and selection of reported results [35]. The overall quality of evidence for each outcome was rated according to the Grading of Recommendations Assessment, Development, and Evaluation (GRADE) guidelines [36]. Considering that only randomized controlled trials (RCTs) were included, each outcome was initially assigned a high quality. Two reviewers (LT and YW) used the GRADE system to evaluate the quality of evidence, and potential disagreements were resolved through discussions with a third reviewer (JF). Table S2 in Multimedia Appendix 2 presents the GRADE downgrading details.

### Data Synthesis and Analysis

Due to the expected heterogeneity among the studies, we used a random-effects model to conduct a pooled estimate of the postintervention effects. Using the inverse-variance method, we calculated the standardized mean differences (SMDs) and 95% CIs for continuous variables. The effect sizes were interpreted as small (0.2), medium (0.5), or large (0.8). Statistical heterogeneity was evaluated using *I*^2^, which was classified as low (*I*^2^<25%), moderate (*I*^2^=25%-50%), high (*I*^2^=50%-75%), and considerably high (*I*^2^>75%). Publication bias was assessed by calculating Begg and Egger test values, and *P*<.05 was considered publication bias [37,38]. A leave-one-out sensitivity analysis was performed to explore the robustness of the pooled effect [39]. We performed all statistical analyses using Review Manager (version 5.4; Cochrane) and Stata (version 17; StataCorp LLC).

## Results

### Compliance With the Registered Protocol

In the initial stage of the research design, our focus was on the impact of remote web-based interventions on physiological states (pain and fatigue) and the quality of life of patients with cancer. When we extracted the relevant data from the included studies, we found that there were controversies regarding the efficacy of remote web-based interventions on the anxiety and depression of patients with cancer. Although anxiety and depression were not listed as outcome indicators in the initial registration protocol, they are closely related to the research topic. Considering that anxiety and depression are common indicators of the psychological state of patients with cancer and have a significant impact on their lives, anxiety and depression cannot be ignored when evaluating the effect of remote web-based interventions. Therefore, to enrich the research content and enhance the clinical significance of the results, compared with the registered protocol, we have added outcome indicators of anxiety and depression. There were no other inconsistencies with the preregistration protocol.

### Study Selection

**Figure 1:**
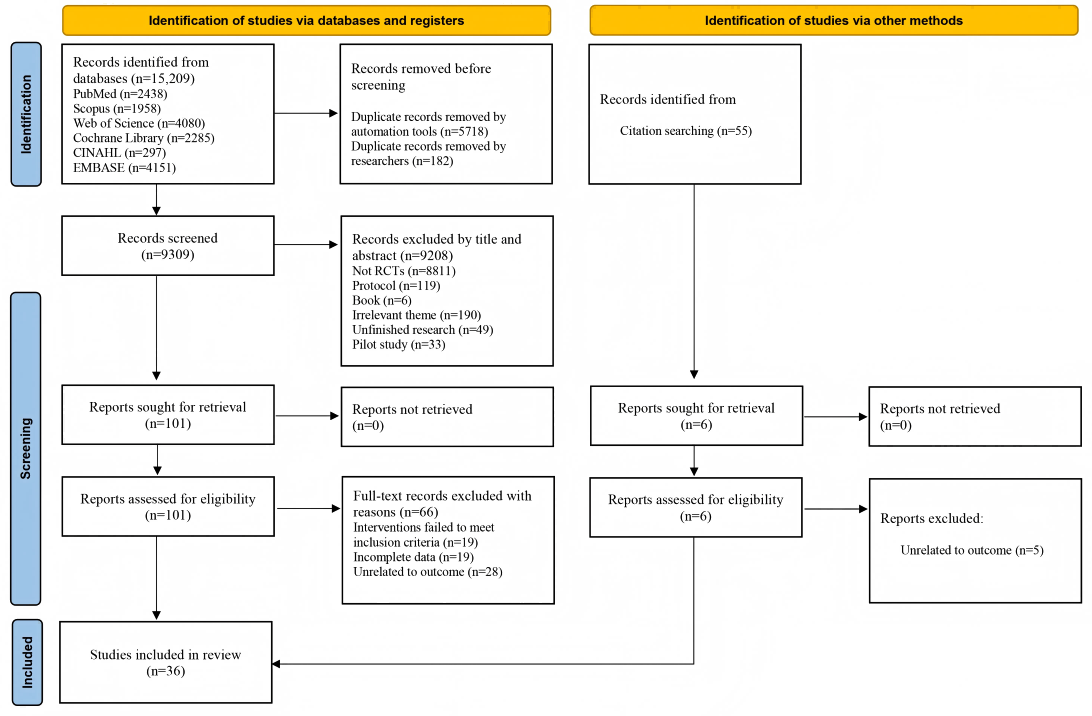
PRISMA flowchart for study selection. RCT: randomized controlled trial.

Figure 1 illustrates the flow of this study and the reasons for exclusion. A total of 15,209 records were identified from the databases. After removing duplicate literature, 9309 (61.2%) potentially eligible studies were determined. After screening the titles and abstracts, we retrieved 101 studies for a more in-depth review. Eventually, 35 (34.6%) RCTs met the inclusion criteria. Among the 66 excluded studies, 19 (29%) had intervention methods that did not meet the inclusion criteria, 19 (29%) lacked complete data, and 28 (42%) did not contain relevant outcome indicators. In addition, we tracked and supplemented relevant citations. Finally, 36 studies met the inclusion criteria for our meta-analysis [15,19-33,40-59].

### Study Characteristics

A total of 36 RCTs were included in this study [15,19-33,40-59]. The study sites involved countries such as China, Iran, the United States, Spain, the Netherlands, Japan, the United Kingdom, Australia, and Germany. The sample size of the studies varied from 29 to 417, with 4521 patients with cancer included. The age range of patients with cancer was wide (>18 years). The patients with cancer covered various types of cancers, including non–small cell lung cancer, breast cancer, urinary system cancers, digestive system cancers, acute leukemia, esophageal cancer, ovarian cancer, cervical cancer, uterine cancer, vaginal cancer, and mixed cancer types. The apps, miniprograms, or other web-based platforms used in the intervention group included WeChat, WhatsApp, ePAL, Microsoft Teams, Zoom, KLIK Pijnmonitor, Welby My Carte ONC, ASyMS, MyBreastHealth, Kanker Nazorg Wijzer, Breast Cancer Support Zone program, the miniprogram “Electronic Intestinal Safety,” e-CUIDATE system, Breast Cancer Self-Management Support app, ENCOURAGE project, Steps Toward Improving Diet and Exercise website, the website named “Together,” Shafayar, Mika, and Untire mobile app. The intervention duration ranged from 1 week to 18 months, and most of the studies focused on 3 to 12 months. The outcome assessment indicators included anxiety, depression, pain, fatigue, and quality of life. More information about the main results of each study is provided in Table 1.

**Table 1:** Characteristics of the included studies (randomized controlled trials).

Study and year	Country	Participants					Intervention time		Intervention methods			Main outcomes (assessments)		Main results	
		Sample size, n	Age (y), mean (SD)	Sex, n (%)	Types of cancer			Remote web-based intervention group^a^		Control group			Remote web-based intervention group, mean (SD)		Control group, mean (SD)
Sui et al [47], 2020	China	200	IG^b^: 61.37 (11.21); CG^c^: 62.35 (9.98)	Male: 164 (82); female: 36 (18)	Non–small cell lung cancer	12 mo		WeChat		Usual care	Anxiety (HADS^d^); depression (HADS); quality of life (EORTC QLQ-C30^e^)		Anxiety: 5 (2.84); depression: 5.22 (2.77); quality of life: 74.44 (12.06)		Anxiety: 6.69 (4.01); depression: 6.55 (3.42); quality of life: 70.26 (17.29)
Peng et al [27], 2020	China	302	IG: 55.6 (6.8); CG: 56.3 (7)	Male: 144 (47.7); female: 158 (52.3)	Not specified	3 d		WeChat		Usual care	Pain (NRS^f^); anxiety (GAD-7^g^); depression (PHQ-9^h^); quality of life (not specified)		Pain: 2.5 (1.2); anxiety: 9.8 (2.2); depression: 10.1 (2.8); quality of life: 46.3 (5.5)		Pain: 2.8 (1.1); anxiety: 12.4 (3.1); depression: 11.2 (2.2); quality of life: 43.4 (5.2)
Bandani-Susan [57], 2022	Iran	38	IG: 46.79 (12.28); CG: 45.89 (7.64)	Male: 0 (0); female: 38 (100)	Breast cancer	7 wk		WhatsApp		Usual care	Fatigue (CFS^i^)		Fatigue: 26.52 (10.32)		Fatigue: 28.16 (9.82)
Xu et al [44], 2021	China	126	IG: 48.74 (6.88); CG: 47.13 (7.58)	Male: 0 (0); female: 126 (100)	Breast cancer	9 mo		WeChat		Usual care	Quality of life (FACT-B^j^)		Quality of life: 84.39 (8.54)		Quality of life: 72.11 (8.85)
Hao et al [52], 2022	China	80	IG: 64.8 (12.58); CG: 63.25 (17.16)	Not specified	Urinary system cancers (prostate, testicular, penile, kidney, and ureter, bladder)	3 mo		WeChat		Usual care	Quality of life (EORTC QLQ-C30)		Quality of life: 74.63 (4.25)		Quality of life: 67.20 (3.26)
Zhao et al [21], 2024	China	42	51-75^k^	Male: 23 (55); female: 19 (45)	Multiple cancers	4 wk		WeChat		Usual care	Pain (NRS)		Pain: 1.86 (0.79)		Pain: 2.76 (1)
Wang et al [28], 2022	China	72	≥18^k^	Male: 40 (55); female: 32 (45)	Acute leukemia	12 wk		WeChat		Usual care	Anxiety (HADS); depression (HADS); quality of life (FACT-Leu^l^); fatigue (BFI-C^m^)		Anxiety: 11.00 (1.72); depression: 12.88 (2.21); quality of life: 65.34 (6.6); fatigue: 2.51 (0.54)		Anxiety: 16.29 (2.16); depression: 16.29 (2.98); quality of life: 71.55 (9.2); fatigue: 4.62 (1.34)
Wang et al [22], 2024	China	125	IG: 40.29 (19.73); CG: 40.53 (22)	Male: 0 (0); female: 125 (100)	Breast cancer	6 mo (24 wk)		WeChat		Usual care	Pain (NRS); quality of life (FACT-B); fatigue (NRS)		Pain: 1.72 (1.76); quality of life: 108.69 (13.58); fatigue: 2.36 (1.46)		Pain: 2.86 (2.12); quality of life: 93.28 (13.01); fatigue: 1.97 (2.39)
Zheng et al [43], 2022	China	150	Trial 1—IG: 57.48 (9.29) and CG: 58.46 (9.96); Trial 2—IG: 59.50 (10.67) and CG: 58.46 (9.96)	Male: 123 (82); female: 27 (18)	Digestive system cancers (colon, rectum, stomach, liver, and esophagus)	4 wk		WeChat		Usual care	Anxiety (HADS); depression (HADS)		Trial 1—anxiety: 2.84 (2.61) and depression: 2.86 (2.8); Trial 2—anxiety: 2.68 (2.55) and depression: 3.32 (3.05)		Trial 1—anxiety: 4.5 (4.06) and depression: 4.58 (4.39); Trial 2—anxiety: 4.50 (4.06) and depression: 4.58 (4.39)
Yu et al [20], 2022	China	168	≥18^k^	Male: 139 (83); female: 29 (17)	Esophageal cancer	6 mo		Telephone and internet		Usual care	Pain (EORTC QLQ-C30); quality of life (EORTC QLQ-C30); fatigue (EORTC QLQ-C30)		Pain: 6.74 (12.74); quality of life: 81.93 (12.58); fatigue: 19.1 (11.24)		Pain: 14.84 (21.91); quality of life: 65.55 (16.76); fatigue: 37.67 (19.58)
Somers et al [24], 2016	United States	30	IG: 60 (11)	Male: 15 (50); female: 15 (50)	Cancers (breast, prostate, lung, and colorectal)	4 sessions over an unspecified period (designed to be weekly)		iPad (Skype)		Usual care	Pain (BPI^n^)		Pain: 3.32 (1.89)		Pain: 2.05 (1.95)
Kamdar et al [31], 2024	United States	112	IG: 54.6 (10.9); CG: 50.7 (12.4)	Male: 57 (51); female: 55 (49)	Multiple cancers	8 wk		ePAL		Usual care	Pain (BPI); anxiety (GAD-7)		Pain: 3.16 (1.78); anxiety: 6.71 (5.07)		Pain: 4.28 (1.99); anxiety: 5.03(3.67)
Bilmiç et al [26], 2023	Turkey	110	≥18^k^	Male: 37 (34); female: 73 (66)	Multiple cancers	4 wk		Microsoft Teams, Zoom, and WhatsApp		Usual care	Pain (BPI); anxiety (ESAS^o^); depression (ESAS); fatigue (ESAS)		Pain: 3.88 (3); anxiety: 4.52 (3.1); depression: 4.04 (3.4); fatigue: 4.77 (2.68)		Pain: 2.04 (2.26); anxiety: 4.2 (3.21); depression: 3.09 (3.05); fatigue: 4.67 (2.85)
Martínez-Miranda et al [19], 2024	Spain	49	IG: 49.21 (5.91); CG: 50 (8.04)	Male: 0 (0); female: 49 (100)	Breast cancer	12 wk		WhatsApp and email		Usual care	Pain (BPI); quality of life (FACT-B)		Pain: 2.39 (1.72); quality of life: 83.62 (14.71)		Pain: 4.85 (1.53); quality of life: 65.5 (13.73)
Simon et al [49], 2024	Netherlands	158	IG: 7.5 (5.1); CG: 7.5 (5.4)	Male: 79 (50); female: 79 (50)	Hematology-oncology, neuro-oncology, and solid tumors	18 mo		KLIK Pijnmonitor		Usual care	Pain (BPI)		Pain: 3.2 (2.1)		Pain: 3.9 (2.8)
Okuyama et al [50], 2024	Japan	125	IG: 63.9 (7.8); CG: 62.7 (7.4)	Male: 1 (0.8); female: 124 (99.2)	Breast cancer	3 mo		Welby My Carte ONC		Usual care	Quality of life (FACT-B)		Quality of life: 106.8 (18.4)		Quality of life: 105.2 (18)
Kearney et al [51], 2009	United Kingdom	112	IG: 55.1 (10.6); CG: 56.9 (10.5)	Male: 26 (23); female 86 (77)	Cancers (breast, lung, and colorectal cancer)	12-16 wk		ASyMS^p^		Usual care	Fatigue (combination scale^q^)		Fatigue: 1.54 (1.11)		Fatigue: 1.82 (1.09)
Visser et al [46], 2018	Netherlands	109	IG: 55.8 (8.3); CG: 57.9 (8.8)	Male: 0 (0); female: 109 (100)	Breast cancer	3 mo		Tablet-based web app		Usual care	Quality of life (EORTC-QLQC30)		Quality of life: 75.28 (16.74)		Quality of life: 73.33 (15.43)
Zhu et al [40], 2018	China	114	IG: 46.2 (8.5); CG: 47.2 (8.3)	Male: 0 (0); female: 114 (100)	Breast cancer	12 wk		The app-based BCS^r^ program		Usual care	Anxiety (HADS); depression (HADS); quality of life (FACT-B)		Anxiety: 9.93 (2.72); depression: 10.28 (2.46); quality of life: 92.87 (21.39)		Anxiety: 10.28 (2.46); depression: 12.58 (2.15); quality of life: 84.09 (15.99)
Zhou et al [42], 2020	China	111	IG: 49.84 (8.85); CG: 49.98 (9.84)	Male: 0 (0); female: 111 (100)	Breast cancer	6 mo		WeChat		Usual care	Pain (NRS); quality of life (FACT-B); fatigue (NRS)		Pain: 1.91 (2.07); quality of life: 106 (11.73); fatigue: 2.33 (2.09)		Pain: 2.71 (1.8); quality of life: 98.42 (16.59); fatigue: 1.75 (1.87)
Xia [45], 2020	China	155	18-70^k^	Male: 83 (54); female: 72 (46)	Low rectal cancer	3 mo		WeChat, blog, telephone, and QQ, among others		Usual care	Pain (Stoma Care Self-Efficacy Scale); anxiety (STAI^s^); quality of life (Stoma Care Self-Efficacy Scale); fatigue (Stoma Care Self-Efficacy Scale)		Pain: 26.59 (6.48); anxiety: 27.21 (6.88); quality of life: 47.59 (9.26); fatigue: 39.50 (3.26)		Pain: 33.75 (7.82); anxiety: 38.02 (6.33); quality of life: 45.84 (8.2); fatigue: 51.86 (4.56)
Chen et al [33], 2024	China	91	IG: 48.09 (8.25); CG: 50.59 (6.10)	Male: 0 (0); female: 91 (100)	Breast cancer	7 wk		WeChat		Usual care	Anxiety (HADS); depression (HADS); quality of life (EORTC-QLQC30)		Anxiety: 7.59 (2.69); depression: 7.5 (2.54); quality of life: 79.15 (5.45)		Anxiety: 10.11 (1.94); depression: 11.11 (2.54); quality of life: 70.97 (7.35)
Balci and Faydali [58], 2024	Turkey	81	IG: 47.04 (10.21); CG: 54.24 (12.92)	Male: 0 (0); female: 81 (100)	Breast cancer	1 wk		App (MyBreastHealth)		Usual care	Quality of life (WHOQOL-BREF^t^)		Quality of life: 72.26 (14.12)		Quality of life: 42.50 (14.38)
Çınar et al [56], 2021	Turkey	64	IG: 45.9 (8.3); CG: 45.5 (9.8)	Male: 0 (0); female: 64 (100)	Breast cancer	12 wk		Mobile app		Usual care	Quality of life (FACT-ES QLS^u^)		Quality of life: 137.58 (16.94)		Quality of life: 123.7 (20)
Willems et al [30], 2017	Netherlands	409	IG: 56.26 (10.84); CG: 56.28 (11.45)	Male: 77 (18.8); female: 332 (81.2)	Multiple cancers	6 mo		Kanker nazorg wijzer (cancer aftercare guide)		Usual care	Anxiety (HADS); depression (HADS); quality of life (EORTC-QLQC30); fatigue (CIS^v^)		Anxiety: 4.39 (3.89); depression: 2.82 (3.06); quality of life: 77.44 (18.17); fatigue: 55.9 (26.72)		Anxiety: 4.66 (3.97); depression: 3.53 (3.68); quality of life: 75.6 (20.58); fatigue: 61.77 (28.15)
Ghanbari et al [53], 2021	Iran	82	IG: 46.9 (9.83); CG: 46 (8.8)	Male: 0 (0); female: 82 (100)	Breast cancer	4 wk		Breast Cancer Support zone		Usual care	Anxiety (STAI)		Anxiety: 90.66 (13.84)		Anxiety: 106.92 (15.94)
Zhou et al [41], 2024	China	120	IG: 62.72 (7.91); CG: 61.78 (11.8)	Male: 79 (66); female: 41 (34)	Rectal cancer	3 mo		“e-bowel safety” applet		Usual care	Quality of life (EORTC-QLQC30)		Quality of life: 83.41 (2.46)		Quality of life: 78.71 (2.72)
Galiano-Castillo et al [54], 2016	Spain	76	IG: 47.4 (9.6); CG: 49.2 ( 7.9)	Male: 0 (0); female: 76 (100)	Breast cancer	8 wk		e-CUIDATE system		Usual care	Pain (BPI); quality of life (EORTC-QLQC30)		Pain: 2.53 (2.16); quality of life: 73.50 (20.76)		Pain: 4.12 (2.13); quality of life: 50.45 (20.78)
Hou et al [15], 2020	China	112	＞20^k^	Male: 0 (0); female: 112 (100)	Breast cancer	3 mo		BCSMS^w^ app		Usual care	Quality of life (EORTC-QLQC30)		Quality of life: 79.13 (15.31)		Quality of life: 79.49 (12.41)
Admiraal et al [59], 2017	Netherlands	125	＞18^k^	Male: 0 (0); female: 125 (100)	Breast cancer	12 wk		ENCOURAGE program		Usual care	Quality of life (EORTC-QLQC30)		Quality of life: 74.33 (19.42)		Quality of life: 73.41 (17.67)
Dong et al [23], 2019	China	50	IG: 48 (5.54); CG: 51.63 (7.49)	Male: 0 (0); female: 50 (100)	Breast cancer	12 wk		Phone step-recording app, social media apps		Usual care	Pain (SF-36^x^); quality of life (SF-36)		Pain: 77.31 (13.43); quality of life: 73.38 (18.16)		Pain: 79.58 (16.28); quality of life: 63.08 (18.9)
Frensham et al [25], 2018	Australia	91	IG: 65.2 (9.3); CG: 66.1 (9.4)	Male: 44 (48); female: 47 (52)	Multiple cancers	13 wk		STRIDE^y^ website		Usual care	Pain (SF-36v2^z^); quality of life (SF– 36v2)		Pain: 63.3 (18.5); quality of life: 69.5 (16.5)		Pain: 61.6 (20.3); quality of life: 67.4 (15.7)
Petzel et al [29], 2018	United States	29	IG: 59.6 (10); CG: 55.5 (8.4)	Male: 0 (0); female: 29 (100)	Ovarian cancer	2 mo		A website named “Together”		Usual care	Anxiety (HADS); depression (HADS)		Anxiety: 7.0 (3.6); depression: 4.9 (3.9)		Anxiety: 7.8 (2.7); depression: 3.2 (2.9)
Doosti et al [55], 2024	Iran	68	IG: 49.41 (8.8); CG: 50.26 (13.64)	Male: 0 (0); female: 68 (100)	Cancers (ovarian, cervical, uterus, and vaginal)	8 wk		Shafayar		Usual care	Anxiety (STAI); quality of life (EORTC-QLQC30)		Anxiety: 47.23 (12.38); quality of life: 73.78 (16.79)		Anxiety: 57.41 (12.72); quality of life: 62.41 (16.62)
Springer et al [32], 2024	Germany	218	IG: 55 (11); CG: 58 (10)	Male: 132 (61); female: 86 (39)	Multiple cancers	12 wk		Mika		Usual care	Anxiety (HADS); depression (HADS); quality of life (CGI-I^aa^); fatigue (FACIT-F^ab^)		Results 1 (ITT^ac^)—anxiety: 7.7 (4.3); depression: 6.4 (4.5); quality of life: 3.8 (1.1); fatigue: 32.2 (10.8) and Results 2 (PP^ad^)—anxiety: 7 (4.3); depression: 5.6 (4.4); quality of life: 3.7 (1.2); fatigue: 34.2 (11.4)		Results 1 (ITT)—anxiety: 8.2 (4.1); depression: 7.5 (4.9); quality of life: 3.9 1.4); fatigue: 31.2 (13) and Results 2 (PP)—anxiety: 7.8 (4); depression: 7.1 (4.7); quality of life: 4.1 (1.3); fatigue: 32.9 (12.8)
Spahrkäs et al [48], 2020	Australia, Canada, United Kingdom, and United States	417	IG: 56.7 (9.99); CG: 56.2 (9.42)	N/A^ae^	Multiple cancers	12 wk		Untire mobile app		Usual care	Quality of life (EORTC QLQ-30); fatigue (FSI^af^)		Quality of life: 4.44 (1.27); fatigue: 5.81 (1.74)		Quality of life: 4.45 (1.14); fatigue: 6.2 (1.57)

^a^Remote web-based intervention group: Intervention methods were based on remote methods, such as relevant apps, applets, and websites, among others.

^b^IG: intervention group.

^c^CG: control group.

^d^HADS: Hospital Anxiety and Depression Scale.

^e^EORTC QLQ-C30: European Organization for Research and Treatment of Cancer Quality of Life Questionnaire Core 30.

^f^NRS: Numerical Rating Scale.

^g^GAD-7: Generalized Anxiety Disorder-7.

^h^PHQ-9: Patient Health Questionnaire-9.

^i^CFS: Cancer Fatigue Scale.

^j^FACT-B: Functional Assessment of Cancer Therapy—Breast.

^k^Data reported as range.

^l^FACT-Leu: the Chinese version of the Functional Assessment of Cancer Therapy.

^m^BFI-C: the Chinese version of the Brief Fatigue Inventory-Leukemia.

^n^BPI: Brief Pain Inventory.

^o^ESAS: Edmonton Symptom Assessment Scale.

^p^ASyMS: Advanced Symptom Management System.

^q^Combination Scale^:^ The scale is a symptom questionnaire that integrates the Common Terminology Criteria for Toxicity of Adverse Events grading system and the Chemotherapy Symptom Assessment Scale.

^r^BCS: breast cancer electronic support.

^s^STAI: State-Trait Anxiety Inventory.

^t^WHOQOL-BREF: World Health Organization Quality of Life Scale-Brief Version.

^u^FACT-ES QLS: Functional Assessment of Cancer Therapy—Endocrine Symptoms Quality of Life Scale.

^v^CIS: Checklist Individual Strength.

^w^BCSMS: Breast Cancer Self-Management Support.

^x^SF-36: 36-item Short Form Health Survey.

^y^STRIDE: Steps Toward Improving Diet and Exercise.

^z^SF-36v2: Short Form (36) Health Survey, version 2.

^aa^CGI-I: Clinical Global Impression—Improvement Scale.

^ab^FACIT-F: Functional Assessment of Chronic Illness Therapy—Fatigue.

^ac^ITT: intention-to-treat.

^ad^PP: per protocol.

^ae^N/A: not applicable.

^af^FSI: Fatigue Symptom Inventory.

### Risk of Bias

As shown in Figure S1 in Multimedia Appendix 2 [15,19-33,40-59], a total of 25 (69%) studies were assessed as having an overall low risk [15,20-23,25-27,32,33,40-45, 47,48,50,52,54-56,58,59], 7 (19%) studies were assessed as having some concerns [19,28-31,49,51], and 4 (11%) studies were assessed as having a high risk [24,46,53,57]. Except for 1 (3%) study [46], which was assessed as having a high risk, the remaining 35 (97%) studies reported an adequate randomization process and were evaluated as having a low risk of bias. Regarding deviation from the intended interventions, 35 (97%) studies were considered to have a low risk of bias, and 1 (3%) study had some concerns [49]. Regarding missing outcome data, 28 (78%) studies were rated as having a low risk of bias, 6 (17%) had some concerns [19,28-31,51], and another 2 (5%) were assessed as having a high risk of bias [24,57]. The outcome measurement risks of 2 (5%) studies were rated as having a high risk of bias [53,57], and the remaining 34 (94%) studies were rated as having a low risk of bias. Regarding the selection of reported results, 36 (100%) studies had a low risk of bias. More detailed information on the risk-of-bias assessment is provided in Table S3 in Multimedia Appendix 2 [15,19-33,40-59].

### Outcomes From Included Studies

#### Overview

By including 36 eligible RCTs, we conducted a systematic review and meta-analysis to examine the efficacy of remote web-based interventions on pain (n=14, 39% studies [19-27,31,42,45,49,54]), fatigue (n=11, 31% studies [20,22,26,28,30,32,42,45,48,51,57]), anxiety (n=14, 39% studies [26-33,40,43,45,47,53,55]), depression (n=10, 28% studies [26-30,32,33,40,43,47]), and quality of life (n=26, 72% studies [15,19,20,22,23,25,27,28,30,32,33,40-42,44-48,50,52,54-56,58,59]) in patients with cancer. Physiologically, the meta-analysis results showed that remote web-based interventions could significantly reduce the pain intensity and relieve the fatigue state of patients with cancer. Regarding psychological state, remote web-based interventions had a substantial effect on the anxiety and depression states of patients with cancer. Overall, remote web-based interventions significantly improved the quality of life of patients with cancer. The specific results of the statistical analysis are described in subsequent sections.

#### Pain

**Figure 2:**
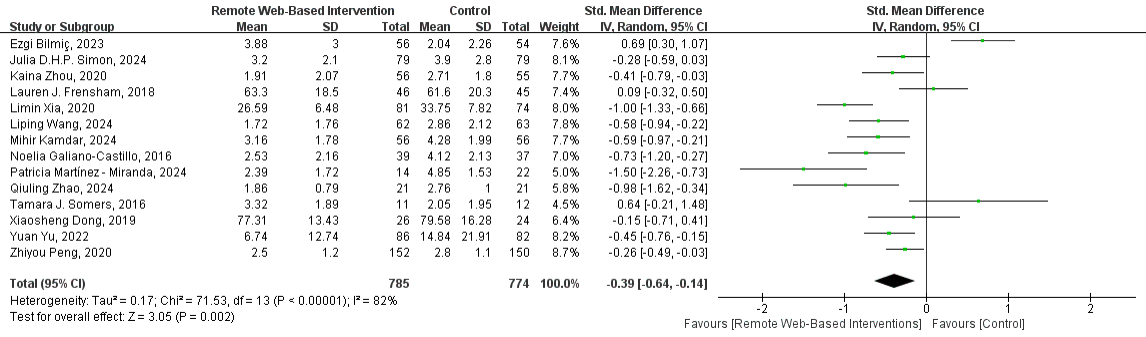
Forest plot for the efficacy of a remote web-based intervention on pain.

As shown in Figure 2 [19-27,31,42,45,49,54], the results of the meta-analysis of 14 (39%) studies indicated that, compared with routine care, remote web-based interventions significantly reduced the pain intensity in patients with cancer (SMD –0.39, 95% CI –0.64 to –0.14; *I*^2^=82%; GRADE rating=low).

#### Fatigue

The results of the meta-analysis of 11 (31%) studies (Figure S2 in Multimedia Appendix 2 [20,22,26,28,30,32,42,45,48,51,57]) showed that remote web-based interventions had a significant effect on the fatigue state of patients with cancer (n=11, 31% studies; SMD –0.52, 95% CI –0.95 to –0.09; *I*^2^=95%; GRADE rating=low).

#### Anxiety

**Figure 3:**
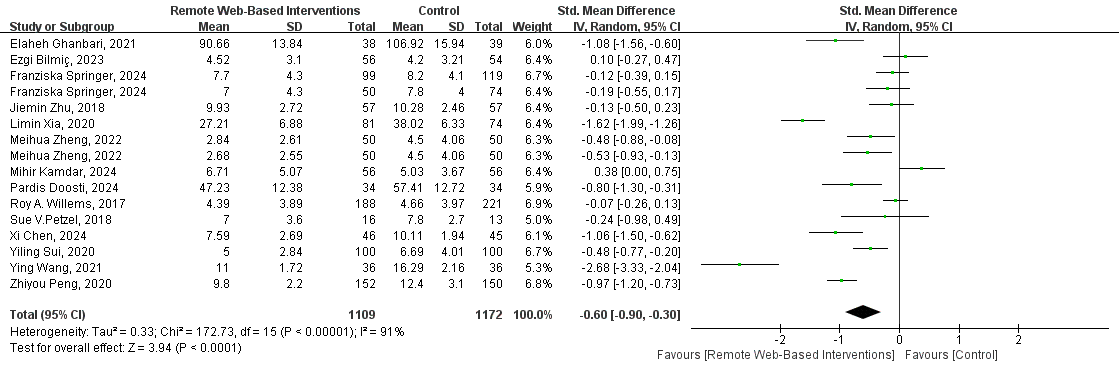
Forest plot for the efficacy of a remote web-based intervention on anxiety.

As shown in Figure 3 [26-33,40,43,45,47,53,55], the results of the meta-analysis of 14 (39%) studies indicated that remote web-based interventions significantly improved the anxiety state of patients with cancer (SMD –0.60, 95% CI=–0.90 to –0.30; *I*^2^=91%; GRADE rating=low).

#### Depression

**Figure 4:**
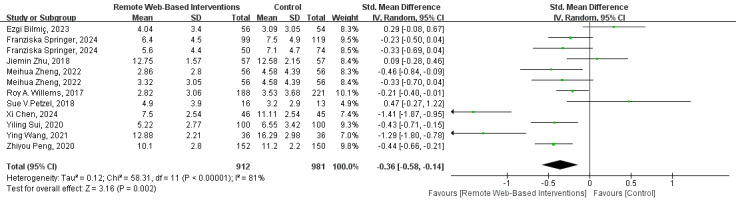
Forest plot for the efficacy of a remote web-based intervention on depression.

The results of the meta-analysis of 10 (28%) studies (Figure 4 [26-30,32,33,40,43,47]) showed that remote web-based interventions could effectively improve the depressive state of patients with cancer (SMD –0.36, 95% CI=–0.58 to –0.14; *I*^2^=81%; GRADE rating=low).

#### Quality of Life

**Figure 5:**
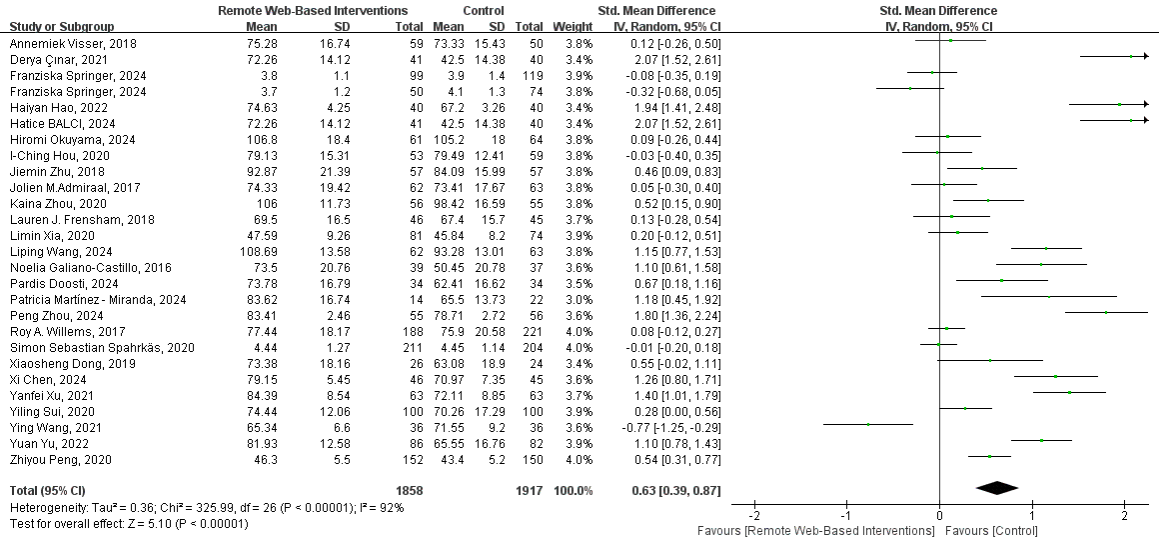
Forest plot for the efficacy of a remote web-based intervention on quality of life.

As shown in Figure 5 [15,19,20,22,23,25,27,28,30,32,33,40-42, 44-48,50,52,54-56,58,59], the results of the meta-analysis of 26 studies indicated that, compared with routine care, remote web-based interventions had a significant positive impact on the quality of life of patients with cancer (SMD 0.63, 95% CI 0.39-0.87; *I*^2^=92%; GRADE rating=low).

### Subgroup Analysis

#### Overview

As shown in Table 2, we conducted subgroup analyses on outcome indicators, such as pain, fatigue, anxiety, depression, and quality of life (region of the country, whether WeChat-based intervention was used, and duration of the intervention). Detailed subgroup analyses of fatigue, anxiety, depression, and quality of life are presented in Tables S4-S7 in Multimedia Appendix 2.

**Table 2:** Subgroup analysis—pain.

Subgroup categories and names			Pooled effect size, SMD^a^ (95% CI)		Heterogeneity (*I*^2^), %
**Region**					
	Asia (n=7)	–0.53 (–0.76 to –0.30)		65	
	America (n=2)	–0.04 (–1.23 to 1.16)		85	
	Europe (n=3)	–0.75 (–1.36 to –0.15)		79	
**App**					
	WeChat (n=4)	–0.40 (–0.57 to –0.24)		46	
	Non-WeChat (n=10)	–0.33 (–0.69 to 0.03)		86	
**Intervention time**					
	<3 mo (n=5)	–0.35 (–0.86 to 0.16)		89	
	>3 mo (n=8)	–0.46 (–0.71 to –0.22)		68	

^a^SMD: standardized mean difference.

#### Pain Subgroup by Country

In the Asian region, the meta-analysis results of 7 (19%) studies [20-23,27,42,45] showed that the pooled effect size SMD was –0.53 (95% CI –0.76 to –0.30), with a heterogeneity of 65%. In the American region, the results of 2 (6%) studies [24,31] showed that the pooled effect size SMD was –0.04 (95% CI –1.23 to 1.16), with a heterogeneity of 85%. In the European region, the results of 3 (8%) studies [19,49,54] showed that the pooled effect size SMD was –0.75 (95% CI –1.36 to –0.15), with a heterogeneity of 79%, suggesting that remote web-based interventions might have a pain-relieving effect in the European region.

#### Pain Subgroup by App

In the WeChat group, the meta-analysis of 4 (11%) studies [21,22,27,42] showed a pooled effect size SMD of –0.40 (95% CI –0.57 to –0.24), with a heterogeneity of 46%. This result indicated that the intervention using WeChat had a certain impact on pain, and the heterogeneity among studies was moderate. In the non-WeChat group, the meta-analysis of 10 (28%) studies [19,20,23-26,31,45,49,54] showed a pooled effect size SMD of –0.33 (95% CI –0.69 to 0.03), with a heterogeneity of 86%.

#### Pain Subgroup by Intervention Duration

In the subgroup with an intervention duration of less than 3 months, the meta-analysis of 5 (14%) studies [21,26,27,31,54] showed a pooled effect size SMD of –0.35 (95% CI –0.86 to 0.16), with a heterogeneity of 89%. In the subgroup with an intervention duration of more than 3 months, the results of 8 (22%) studies [19,20,22,23,25,42,45,49] showed a pooled effect size SMD of –0.46 (95% CI –0.71 to –0.22), with a heterogeneity of 68%, suggesting that a longer-term intervention had a certain pain-relieving effect.

### Sensitivity Analysis

The sensitivity analysis for the meta-analysis results of pain (Table 3), anxiety (Table S8 in Multimedia Appendix 2 [26-33,40,43,45,47,53,55]), depression (Table S9 in Multimedia Appendix 2 [26-28,30,32,33,40,43,47]), and quality of life (Table S10 in Multimedia Appendix 2 [15,19,20,22,23,25, 27,28,30,32,33,40-42,44-48,50,52,54-56,58,59]) showed that when any single study was excluded, the pooled estimates of the main analysis were robust. However, for the meta-analysis result of fatigue, the sensitivity analysis indicated that after excluding the studies by Xia [45] and Wang et al [28], respectively, the results of the overall pooled analysis changed from statistically significant (SMD –0.52, 95% CI –0.95 to –0.09; *I*^2^=95%) to nonstatistically significant (SMD –0.28, 95% CI –0.57 to –0.01; *I*^2^=91% and SMD –0.39, 95% CI –0.81 to –0.03; *I*^2^=91%), suggesting that the main analysis was not robust in the pooled estimates (Table S11 in Multimedia Appendix 2 [20,22,26,28,30,32,42,45,48,51,57]).

**Table 3:** Sensitivity analysis (leave-one-out method)—pain.

Study removed	Pooled effect size after removing this study, SMD^a^ (95% CI)	Heterogeneity (*I*^2^), %
Bilmiç et al [26]	–0.48 (–0.68 to –0.27)	70
Simon et al [49]	–0.40 (–0.67 to –0.12)	83
Zhou et al [42]	–0.39 (–0.66 to –0.12)	83
Frensham et al [25]	–0.43 (–0.69 to –0.17)	82
Xia [45]	–0.33 (–0.58 to –0.09)	79
Wang et al [22]	–0.37 (–0.64 to –0.10)	83
Kamdar et al [31]	–0.37 (–0.64 to –0.10)	83
Galiano-Castillo et al [54]	–0.36 (–0.63 to –0.10)	83
Martínez-Miranda et al [19]	–0.33 (–0.58 to –0.09)	81
Zhao et al [21]	–0.35 (–0.61 to –0.10)	82
Somers et al [24]	–0.44 (–0.69 to –0.19)	82
Dong et al [23]	–0.41 (–0.67 to –0.14)	83
Yu et al [20]	–0.38 (–0.66 to –0.11)	83
Peng et al [27]	–0.40 (–0.69 to –0.12)	83

^a^SMD: standardized mean difference.

### Publication Bias

As shown in Table 4, the results of Begg and Egger tests (*P*＞.05) indicate no publication bias in the pooled analyses of pain, fatigue, anxiety, depression, and quality of life.

**Table 4:** Begg and Egger test values.

Clinical manifestations	Egger test		Begg test	
	Student t-statistic	*P* value	Standard Normal Z-statistic	*P* value

Pain	–0.29	.78	0.22	.83
Fatigue	–1.26	.23	0.75	.45
Anxiety	–1.48	.16	1.4	.16
Depression	–0.51	.62	0.34	.73
Quality of life	1.23	.25	0.187	.06

## Discussion

### Overview

In recent years, the health management of patients with cancer has shifted from single-disease treatment to a holistic focus on both physiological and psychological states. Patients with cancer not only have to cope with physiological symptoms (pain and fatigue) brought about by chemotherapy, radiotherapy, and other treatments, but they are also often burdened with psychosocial issues, including anxiety, depression, posttraumatic stress disorder, and fear of recurrence [60-63]. Although traditional face-to-face interventions can partially alleviate these problems, their accessibility and sustainability are limited due to the uneven distribution of medical resources, patients’ limited mobility, or remote areas [64,65]. Remote web-based interventions, with their advantages of high accessibility, great flexibility, and low cost, have gradually become an important means for patients with cancer to manage their symptoms by themselves [14]. However, although previous studies have suggested that remote web-based interventions may improve physiological and psychological states and quality of life in patients with cancer, the effectiveness remains controversial [19-25,27-33]. To this end, this systematic review and meta-analysis aimed to summarize the relevant evidence regarding the physiological and psychological impacts of remote web-based interventions on patients with cancer. A total of 36 studies were included to determine the clinical efficacy of remote web-based interventions on physiological pain, fatigue, anxiety, depression, and quality of life in patients with cancer.

### The Effect of Remote Web-Based Interventions on Pain

This study, through meta-analysis, found that remote web-based interventions significantly reduced the pain intensity in patients with cancer (n=14, 39% studies; SMD –0.39, 95% CI –0.64 to –0.14; *I*^2^=82%; GRADE rating=low). This result is consistent with the results of some previous studies. For example, in the review by Villinger et al [66], it was mentioned that some apps showed certain positive effects in cancer pain management. Through the real-time chat consultation function, patients can report pain and receive suggestions, which is helpful for pain management. The review by Wu et al [67] also pointed out that mHealth apps have certain potential in monitoring and managing cancer pain. After some patients used pain-related apps, their physiological pain intensity improved. Through remote monitoring and digital platforms, patients with cancer can report symptoms (pain) in real time, enabling health care providers to adjust treatment plans promptly [68]. Meanwhile, the health education module enhances the understanding of relevant symptoms in patients with cancer and improves their self-management [69]. In this study, the pooled effect value of pain showed high heterogeneity, with an overall heterogeneity of 82%. Further subgroup analyses revealed the impacts of different regions, app types, and intervention durations on heterogeneity: the heterogeneity in the Asian subgroup was 65%, 85% in the American subgroup, and 79% in the European subgroup. Regarding the type of intervention app, the heterogeneity in the WeChat subgroup was 46%, while it was 86% in the non-WeChat subgroup. In addition, the heterogeneity in the subgroup with an intervention duration of less than 3 months was 89%, and it decreased to 68% in the subgroup with an intervention duration of more than 3 months. The above results indicate that differences in intervention apps may be one of the main sources of heterogeneity. In contrast, factors such as region and intervention duration are related to heterogeneity but are not the main sources. The sensitivity analysis showed that when any single study was excluded, the main analysis was robust in the pooled estimates, indicating that the study’s results regarding pain have certain reliability.

### The Effect of Remote Web-Based Interventions on Fatigue

Studies show that, in addition to symptom management and educational empowerment [68,69], remote web-based interventions can also customize exercise programs (Tai Chi and aerobic training) for patients with cancer to improve their cardiopulmonary function and muscle strength and alleviate fatigue [70]. Our results indicated that remote web-based interventions significantly affected the fatigue state of patients with cancer (n=11, 31% studies, SMD –0.52, 95% CI –0.95 to –0.09; *I*^2^=95%; GRADE rating=low). However, the sensitivity analysis showed that after excluding the studies by Xia [45] and Wang et al [28], respectively, the statistical significance of the pooled analysis results changed. This result suggests that the main analysis was not robust in the pooled estimates. These 2 studies might have been unique regarding intervention measures, sample characteristics, or fatigue assessment methods, significantly influencing the overall results. The results of the sensitivity analysis indicate that there is still a high degree of uncertainty in current research on the impact of remote web-based interventions on fatigue state of patients with cancer, and this result should be interpreted with caution.

### The Effect of Remote Web-Based Interventions on Anxiety

This study found that remote web-based interventions significantly improved the anxiety state of patients with cancer. This result is consistent with some of the research results mentioned in the review by Villinger et al [66]. Some apps may help relieve patients’ anxiety by providing information support and social interaction functions. Remote web-based intervention can continuously provide psychological support for patients with cancer [71] and comprehensive, personalized support through multidisciplinary collaboration [64]. In addition, the web-based community established by remote web-based interventions can reduce loneliness among patients with cancer. Sharing experiences among patients can enhance their sense of hope [72], thereby improving their psychological states. The pooled effect value of anxiety showed a very high degree of heterogeneity, with an overall heterogeneity as high as 91%. Subgroup analyses further revealed the potential sources of heterogeneity: the heterogeneities of the Asian subgroup, the American subgroup, and the European subgroup were 88%, 54%, and 0%, respectively, indicating significant differences among regions. Regarding the app type, the heterogeneity of the WeChat subgroup was 90%, and that of the non-WeChat subgroup was 74%. Regarding the intervention duration, the heterogeneity of the subgroup with a duration of less than 3 months was 87%, while that of the subgroup with a duration of more than 3 months was 95%. These findings suggest that the high heterogeneity may stem from regional differences and other unknown factors, and the intervention duration and the type of intervention app are not the main sources of high heterogeneity. The sensitivity analysis showed that when any single study was excluded, the main analysis was robust in the pooled estimates, indicating that the results of this study have a certain degree of reliability.

### The Effect of Remote Web-Based Interventions on Depression

This study demonstrated that remote web-based interventions could significantly improve the depressive state of patients with cancer. In the review by Samadbeik et al [73], it was pointed out that mHealth interventions can help improve patients’ psychological states. For example, providing psychological support, self-management education, and other content may positively impact depressive symptoms, which is consistent with the results of our study. The overall heterogeneity of the pooled effect value for depression was 81%. In the subgroup analysis, the heterogeneity of the European subgroup was 0%, and the heterogeneities of the other subgroups were all greater than 50%. This result indicates that the region may be one of the main sources of high heterogeneity. At the same time, the intervention duration and the type of intervention app are not the main sources of high heterogeneity. The results of the sensitivity analysis suggest that the results of this study have a certain degree of reliability.

### The Effect of Remote Web-Based Interventions on Quality of Life

Several studies have shown that digital tools can establish a closed-loop intervention model based on the biopsychosocial medical model through mechanisms such as psychosocial support, social connection and peer support, symptom management and educational empowerment, and multidisciplinary collaborative comprehensive interventions [64,68,69,71]. This intervention model can synergistically improve patients’ physiological and psychological states, enhancing their quality of life. For example, some app interventions mentioned by Villinger et al [66] showed positive effects in aspects such as cancer-related symptom management, thereby improving patients’ quality of life. Samadbeik et al [73] also pointed out that mHealth interventions could improve patients’ quality of life by enhancing self-management and promoting healthy lifestyles. The results of our study also showed that remote web-based interventions had a significant positive impact on the quality of life of patients with cancer. The overall heterogeneity of the pooled effect value for quality of life and the heterogeneities of all subgroups exceeded 50%, which meant that region, the type of intervention app, and intervention duration were not the main sources of high heterogeneity. The results of Begg and Egger tests indicate no publication bias in our study. In addition, the sensitivity analysis results showed that our study was robust.

### Comparison With the Published Systematic Reviews and Meta-Analyses

Previously, 4 systematic reviews [66,67,73,74] and 1 meta-analysis [75] have investigated the effects of apps, miniprograms, or other web-based platforms on symptom management in patients with cancer. We compared our study with the aforementioned articles. We found that Zou et al [74] conducted electronic database searches for studies published until December 2021 and included 20 studies (RCTs=14; quasi-experimental nonrandomized trials=4; and pre-post study=2). Villinger et al [66] carried out a comprehensive and systematic literature search in 5 electronic databases in January 2022 and finally included 17 studies (RCTs=5; nonrandomized trials=3; randomized trial study protocols=3; quasi-experimental design study protocols=1; and pre-post studies=5). Li et al [75] conducted electronic database searches for studies published until April 14, 2021, and included 28 studies (all were RCTs). Wu et al [67] conducted electronic database searches for studies published from 2013 to 2023 and included 20 studies (RCTs=4; quasi-experiments=3; prospective observational cohort studies=4; cross-sectional design=1; qualitative design=3; and mixed methods design=5). Samadbeik et al [73] conducted electronic database searches for studies published between January 2010 and February 2021 and included 28 studies (interventional studies=19, including RCTs; feasibility pilot studies=5; descriptive studies=1; qualitative studies=1; developmental studies=1; nonrandomized controlled studies=1; and mixed methods studies=1). In contrast, we conducted electronic database searches for studies published until October 15, 2024, and included 36 studies (RCTs=36). Compared with the studies by Zou et al [74], Villinger et al [66], Li et al [75], Wu et al [67], and Samadbeik et al [73], our study included newer, more voluminous studies, and all of them were RCTs. Regarding statistical methods, Zou et al [74], Villinger et al [66], Wu et al [67], and Samadbeik et al [73] reported results through comprehensive narrative descriptions. In contrast, we conducted meta-analyses on outcome indicators and reported the results based on them. Furthermore, our study conducted subgroup analysis, sensitivity analysis, and publication bias detection. Although the study by Li et al [75] also reported results using meta-analysis, compared with ours, the outcome indicators in their study were rather single and lacked comprehensiveness. Regarding the conclusions, the studies by Zou et al [74], Villinger et al [66], and Samadbeik et al [73] showed that it was impossible to determine the efficacy of web-based interventions based on apps and miniprograms on pain, fatigue, anxiety, depression, and quality of life in patients with cancer. The study by Li et al [75] indicated that web-based interventions could promote the quality of life of patients with cancer. Wu et al [67] demonstrated that mHealth apps were effective and acceptable in supporting the self-management of cancer pain and could provide patients with multimodal interventions to monitor, track, and manage pain. However, we conducted meta-analyses and quantitatively reported our conclusion: For patients with cancer, remote web-based interventions could alleviate pain, relieve fatigue, improve psychological states, such as anxiety and depression, and enhance their quality of life. In addition, we explored the potential mechanisms of remote web-based interventions to improve the physiological and psychological states of patients with cancer, which enriches the content of our research and theoretical basis. Finally, our conclusions are drawn based on subgroup analysis, sensitivity analysis, and publication bias detection. Therefore, our conclusions are more robust and persuasive.

### Limitations and Strengths

#### Limitations

Several limitations need to be considered in this study. First, the heterogeneity of our conclusions was relatively high. Although subgroup analyses were conducted, the sources of heterogeneity were still not well explained. Second, the impact of confounding factors on our research results has not been further clarified. Third, the sensitivity analysis results indicated that the meta-analysis results regarding the impact of remote web-based interventions on fatigue state in patients with cancer were not robust, and we had to interpret this result with caution. Fourth, due to the differences in the follow-up time points of the included literature, our study only explored the short-term effects of remote web-based interventions on the physiological and mental impacts of patients with cancer. It did not investigate the medium- and long-term clinical efficacies. Finally, we only included studies published in English, which might lead to cultural and language biases, thus limiting the general applicability of the meta-analysis results.

#### Strengths

Despite these limitations, our meta-analysis has some significant strengths. First, this systematic review and meta-analysis only included RCTs, strengthening the evidence supporting the physiological and mental impacts of remote web-based interventions on patients with cancer. Second, the results of the sensitivity analyses showed that our main findings were robust and reliable, and the Begg and Egger tests did not reveal publication bias. Third, the literature we included was more up-to-date, larger in quantity, and comprehensive. Fourth, compared with previous narrative reviews, we conducted meta-analyses. Finally, compared with meta-analysis studies with single outcome indicators, we explored more outcome indicators, and our methods and conclusions were more persuasive.

### Implications for Clinical Practice

The results of this study provided valuable references for clinical practice, indicating that remote web-based interventions can serve as a beneficial supplement to the routine care of patients with cancer. Clinical medical staff should fully recognize the potential of remote web-based interventions in improving the symptoms and quality of life of patients with cancer. On the basis of patients’ specific needs and conditions, they should reasonably select and recommend appropriate remote web-based intervention platforms or tools to provide patients with personalized physiological and mental support.

### Conclusions

Remote web-based interventions have significant effects in reducing pain, relieving fatigue, improving anxiety, alleviating depression, and improving the quality of life of patients with cancer. However, we could not draw firm conclusions about these findings due to the evidence’s low overall certainty. In the future, more high-quality, large-sample RCTs are needed to validate the effectiveness of remote web-based interventions further.

## Appendix

Multimedia Appendix 1 PRISMA Checklist.

Multimedia Appendix 2 Supplementary materials.

Multimedia Appendix 3 Characteristics of the included studies (randomized controlled trials) (continued from Table 1).

## Abbreviations

GRADE: Grading of Recommendations Assessment, Development, and Evaluation
PRISMA: Preferred Reporting Items for Systematic Reviews and Meta-Analyses
RCT: randomized controlled trial
SMD: standardized mean difference

## Data Availability

All data generated or analyzed during this study are included in this paper and Multimedia Appendix 2 or available from the corresponding author upon reasonable request.
