# Supplementary material for: Effects of Remote Web-Based Interventions on the Physiological and Psychological States of Patients With Cancer: Systematic Review With Meta-Analysis
Source: JMIR Mhealth Uhealth. 2025 Jun 12;13:e71196. doi: 10.2196/71196 (PMC12203031; doi:10.2196/71196)
Supplement: Multimedia Appendix 2 [file mhealth_v13i1e71196_app2.docx]

**Effects of Remote Web-Based Interventions on the Physiological and Psychological States of Patients With Cancer: Systematic Review With Meta-Analysis**

[Figure S1: Risk of bias summary: Review authors' judgments about the risk of bias item for each included study. 1](#_Toc22136)

[Figure S2: Forest plot for the efficacy of remote online intervention on fatigue. 5](#_Toc4677)

[Table S1. Search strategy and search results: 6](#_Toc3520)

[Table S2-1. Details of risk of bias assessment of included RCTs 7](#_Toc7830)

[Table S2-2. Details of risk of bias assessment of included RCTs 12](#_Toc29296)

[Table S2-3. Details of risk of bias assessment of included RCTs 17](#_Toc29499)

[Table S3: Subgroup Analysis- Fatigue 22](#_Toc3457)

[Table S4: Subgroup Analysis- Anxiety 23](#_Toc29613)

[Table S5: Subgroup Analysis- Depression 24](#_Toc16736)

[Table S6: Subgroup Analysis- Quality of Life 25](#_Toc14868)

[Table S7 : Sensitivity analysis (leave-one-out method) - Fatigue 26](#_Toc17212)

[Table S8 : Sensitivity analysis (leave-one-out method) - Anxiety 28](#_Toc17382)

[Table S9 : Sensitivity analysis (leave-one-out method) - Depression 30](#_Toc19380)

[Table S10 : Sensitivity analysis (leave-one-out method) - Quality of life 32](#_Toc15449)

[Table S11. GRADE assessments for the certainty of evidence of outcomes. 36](#_Toc8683)

# Figure S1: Risk of bias summary: Review authors' judgments about the risk of bias item for each included study.

| **Study ID** | **Remote online intervention group** | **Control group** | **D1** | **D2** | **D3** | **D4** | **D5** | **Overall** |
| --- | --- | --- | --- | --- | --- | --- | --- | --- |
| Bahar Bandani-Susan, 2021 | WhatsApp | Usual care | 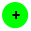 | 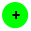 | 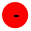 | 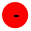 | 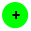 | 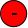 |
| Derya Cinar, 2021 | Mobile app | Usualcare | 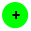 | 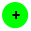 | 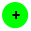 | 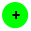 | 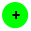 | 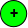 |
| Elaheh Ghanbari, 2021 | Breast Cancer Support zone | Usual care | 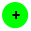 | 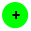 | 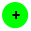 | 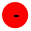 | 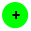 | 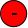 |
| Ezgi Bilmiç, 2023 | Microsoft Teams, Zoom, WhatsApp | Usual care | 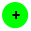 | 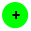 | 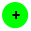 | 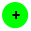 | 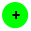 | 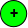 |
| Franziska Springer, 2024 | Mika | Usual care | 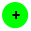 | 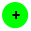 | 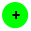 | 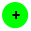 | 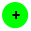 | 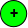 |
| Haiyan Hao, 2022 | WeChat | Usual care | 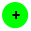 | 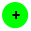 | 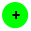 | 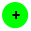 | 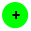 | 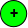 |
| Hatice BALCI, 2024 | app(MyBreastHealth) | Usual care | 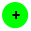 | 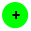 | 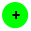 | 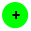 | 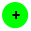 | 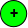 |
| Hiromi Okuyama, 2024 | Welby My Carte ONC! | Usual care | 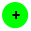 | 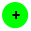 | 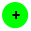 | 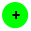 | 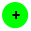 | 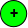 |
| I-Ching Hou, 2020 | BCSMS app | Usual care | 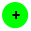 | 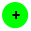 | 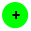 | 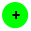 | 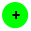 | 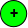 |
| Jolien M.Admiraal, 2017 | Encourage program | Usual care | 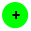 | 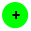 | 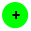 | 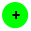 | 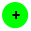 | 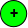 |
| Julia D.H.P.Simon, 2024 | Klik Pijnmonitor | Usual care | 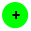 | 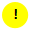 | 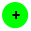 | 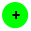 | 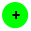 | 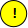 |
| Kaina Zhou, 2020 | WeChat | Usual care | 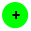 | 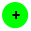 | 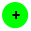 | 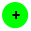 | 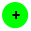 | 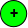 |
| N. Kearney, 2009 | ASyMMS | Usual care | 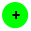 | 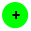 | 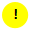 | 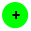 | 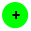 | 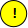 |
| Lauren J.Frensham, 2018 | Stride website | Usual care | 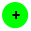 | 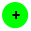 | 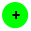 | 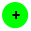 | 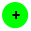 | 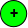 |
| Li-Ping Wang, 2024 | WeChat | Usual care | 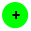 | 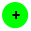 | 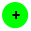 | 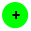 | 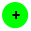 | 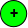 |
| Limin Xia, 2020 | WeChat, blog, telephone, QQ, etc | Usual care | 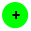 | 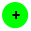 | 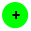 | 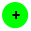 | 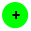 | 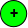 |
| Meihua Zheng, 2022 | WeChat | Usual care | 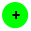 | 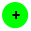 | 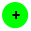 | 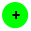 |  |  |
| Mihir Kamdar, 2024 | ePAL | Usual care |  |  |  |  |  |  |
| Noelia Galiano-Castillo, 2016 | e-CUDATE system | Usual care |  |  |  |  |  |  |
| Pardis Doosti, 2024 | Shafayar | Usual care |  |  |  |  |  |  |
| Patricia Martinez-Miranda, 2024 | WhatsApp and Email | Usual care |  |  |  |  |  |  |
| Peng Zhou, 2024 | e-bowel safety applet | Usual care |  |  |  |  |  |  |
| Qiuling Zhao, 2024 | WeChat | Usualcare |  |  |  |  |  |  |
| Roy A. Willems, 2017 | Kanker Nazorg Wijzer | Usual care |  |  |  |  |  |  |
| Simon Sebastian Spahrkäs, 2020 | Untire mobile app | Usual care |  |  |  |  |  |  |
| Sue V.Petzel, 2018 | A website named "Together" | Usual care |  |  |  |  |  |  |
| Tamara J. Somers, 2016 | iPad (skype) | Usual care |  |  |  |  |  |  |
| Anemiek Visser, 2018 | Tablet-based online app | Usual care |  |  |  |  |  |  |
| Xi Chen, 2024 | WeChat | Usual care |  |  |  |  |  |  |
| Xiaosheng Dong, 2019 | Phone step-recording app | Usual care |  |  |  |  |  |  |
| Yanfei Xu, 2021 | WeChat | Usual care |  |  |  |  |  |  |
| Yiling Sui, 2020 | WeChat | Usual care |  |  |  |  |  |  |
| Ying Wang, 2021 | WeChat | Usual care |  |  |  |  |  |  |
| Yuan Yu, 2022 | telephone and Internet | Usual care |  |  |  |  |  |  |
| Zhiyou Peng, 2020 | WeChat | Usualcare |  |  |  |  |  |  |
| Jiemin Zhu, 2018 | The app-based breast cancer e-support (BCS) program | Usual care |  |  |  |  |  |  |

Abbreviations: low risk of bias; high risk of bias; some concerns

Notes: D1, randomization process; D2, deviations from intended intervention; D3, missing outcome data; D4, measurement of outcomes; D5, selection of reported results

# Figure S2: Forest plot for the efficacy of remote online intervention on fatigue.

# Table S1. Search strategy and search results:

| **Database** | **Number of articles** | **Date of search** |
| --- | --- | --- |
| PubMed# | 2438 | October 15, 2024 |
| Scopus | 1958 | October 15, 2024 |
| Web of Science | 4080 | October 15, 2024 |
| Cochrane Library | 2285 | October 15, 2024 |
| CINAHL | 297 | October 15, 2024 |
| EMBASE | 4151 | October 15, 2024 |

Note:

#: The search strategy for PubMed was as follows: ((((((((((("Depression"[Mesh]) OR ("Anxiety"[Mesh])) OR ("Fatigue"[Mesh])) OR ("Quality of Life"[Mesh])) OR ("Pain"[Mesh])) OR (pain)) OR (fatigue)) OR (quality of life)) OR (anxiety)) OR (depression)) AND (("Neoplasms"[Mesh]) OR (cancer OR neoplasms OR Neoplasia OR Neoplasias OR Neoplasm OR Tumors OR Tumor OR Cancer OR Cancers OR Malignancy OR Malignancies))) AND ((((((((((((((((((((((((((((((((((((((("Mobile Applications"[Mesh]) OR (mobile health)) OR (mlearning)) OR (m learning)) OR (mhealth)) OR (m-health)) OR (Mobile Applications)) OR (Smartphone)) OR (handheld computers)) OR (handheld computer)) OR (cellphone)) OR (Cellphones)) OR (Cell phone)) OR (Cell phones)) OR (Cellular phone)) OR (Cellular phones)) OR (Mobile phone)) OR (Mobile phones)) OR (Mobile device)) OR (Mobile devices)) OR (Mobile-based)) OR (Mobile telephone)) OR (Mobile telephones)) OR (mobile health apps)) OR (mobile health application)) OR (mobile health applications)) OR (mobile game)) OR (mobile games)) OR (Smartphone)) OR (Smartphones)) OR (Smart phone)) OR (Smart phones)) OR (Cellular Telephone)) OR (Cellular Telephones)) OR (Telephones AND Cellular)) OR (WeChat)) OR (WhatsApp)) OR (twitter)) OR (facebook)). It was adapted for the other databases.

# Table S2-1. Details of risk of bias assessment of included RCTs

| **Domain** | **Signaling question** | **Response** | | | | | | | | | | | |
| --- | --- | --- | --- | --- | --- | --- | --- | --- | --- | --- | --- | --- | --- |
|  |  | 1. Kearney   (2009) | Lauren J.Frensham  (2018) | Li-Ping Wang  (2024) | Limin Xia  (2020) | eihua Zheng  (2022) | Mihir Kamdar  (2024) | Noelia Galiano-Castillo  (2016) | Pardis Doosti  (2024) | Patricia Martinez-Miranda  (2024) | Peng Zhou  (2024) | Qiuling Zhao  (2024) | Roy A. Willems  (2017) |
| **Bias arising from the randomization process** | 1.1 Was the allocation sequence random? | Y | Y | PY | Y | Y | Y | Y | Y | Y | Y | Y | Y |
|  | 1.2 Was the allocation sequence concealed until participants were enrolled and assigned to interventions? | Y | Y | Y | Y | Y | Y | Y | Y | Y | Y | Y | Y |
|  | 1.3 Did baseline differences between intervention groups suggest a problem with the randomization process? | N | N | N | N | N | PN | N | N | N | N | N | N |
|  | **Risk of bias judgement** | Low | Low | Low | Low | Low | Low | Low | Low | Low | Low | Low | Low |
| **Bias due to deviations from intended interventions** | 2.1.Were participants aware of their assigned intervention during the trial? | Y | Y | Y | Y | Y | Y | Y | Y | Y | Y | Y | Y |
|  | 2.2.Were carers and people delivering the interventions aware of participants' assigned intervention during the trial? | Y | Y | Y | Y | Y | Y | Y | Y | Y | Y | Y | Y |
|  | 2.3. If Y/PY/NI to 2.1 or 2.2: Were there deviations from the intended intervention that arose because of the experimental context? | PN | PN | PN | PN | PN | PN | N | N | N | N | PN | PN |
|  | 2.4 If Y/PY to 2.3: Were these deviations likely to have affected the outcome? | NA | NA | NA | NA | NA | NA | NA | NA | NA | NA | NA | NA |
|  | 2.5. If Y/PY/NI to 2.4: Were these deviations from intended intervention balanced between groups? | NA | NA | NA | NA | NA | NA | NA | NA | NA | NA | NA | NA |
|  | 2.6 Was an appropriate analysis used to estimate the effect of assignment to intervention? | Y | Y | Y | Y | Y | Y | Y | Y | Y | Y | Y | Y |
|  | 2.7 If N/PN/NI to 2.6: Was there potential for a substantial impact (on the result) of the failure to analyse participants in the group to which they were randomized? | NA | NA | NA | NA | NA | NA | NA | NA | NA | NA | NA | NA |
|  | **Risk of bias judgement** | Low | Low | Low | Low | Low | Low | Low | Low | Low | Low | Low | Low |
| **Bias due to missing outcome data** | 3.1 Were data for this outcome available for all, or nearly all, participants randomized? | N | N | Y | Y | PY | N | PY | PY | PY | PY | PY | PY |
|  | 3.2 If N/PN/NI to 3.1: Is there evidence that result was not biased by missing outcome data? | PY | PN | NA | NA | NA | PY | NA | NA | NA | NA | NA | NA |
|  | 3.3 If N/PN to 3.2: Could missingness in the outcome depend on its true value? | NA | PN | NA | NA | NA | NA | NA | NA | NA | NA | NA | NA |
|  | 3.4 If Y/PY/NI to 3.3: Is it likely that missingness in the outcome depended on its true value? | NA | NA | NA | NA | NA | NA | NA | NA | NA | NA | NA | NA |
|  | **Risk of bias judgement** | Some concerns | Low | Low | Low | Low | Some concerns | Low | Low | Some concerns | Low | Low | Some concerns |
| **Bias in measurement of the outcome** | 4.1 Was the method of measuring the outcome inappropriate? | N | N | N | N | N | N | N | N | N | N | N | N |
|  | 4.2 Could measurement or ascertainment of the outcome have differed between intervention groups? | N | N | N | N | N | N | N | N | N | N | N | N |
|  | 4.3 Were outcome assessors aware of the intervention received by study participants? | N | N | N | N | N | N | PY | N | N | N | N | N |
|  | 4.4 If Y/PY/NI to 4.3: Could assessment of the outcome have been influenced by knowledge of intervention received? | NA | NA | NA | NA | NA | NA | N | NA | NA | NA | NA | NA |
|  | 4.5 If Y/PY/NI to 4.4: Is it likely that assessment of the outcome was influenced by knowledge of intervention received? | NA | NA | NA | NA | NA | NA | NA | NA | NA | NA | NA | NA |
|  | **Risk of bias judgement** | Low | Low | Low | Low | Low | Low | Low | Low | Low | Low | Low | Low |
| **Bias in selection of the reported result** | 5.1 Were the data that produced this result analysed in accordance with a pre-specified analysis plan that was finalized before unblinded outcome data were available for analysis? | Y | Y | Y | Y | Y | Y | Y | Y | Y | Y | Y | Y |
|  | 5.2 ... multiple eligible outcome measurements (e.g. scales, definitions, time points) within the outcome domain? | N | N | N | N | N | N | N | N | N | N | N | N |
|  | 5.3 ... multiple eligible analyses of the data? | N | N | N | N | N | N | N | N | N | N | N | N |
|  | **Risk of bias judgement** | Low | Low | Low | Low | Low | Low | Low | Low | Low | Low | Low | Low |
| **Overall bias** | **Risk of bias judgement** | Some concerns | Low | Low | Low | Low | Some concerns | Low | Low | Some concerns | Low | Low | Some concerns |

***Notes: ‘Y’ indicates yes, ‘PY’ indicates probably yes, ‘PN’ indicates probably no, ‘N’ indicates no, ‘NI’ indicates no information, ‘NA’ indicates not applicable.***

# Table S2-2. Details of risk of bias assessment of included RCTs

| **Domain** | **Signaling question** | **Response** | | | | | | | | | | | |
| --- | --- | --- | --- | --- | --- | --- | --- | --- | --- | --- | --- | --- | --- |
|  |  | Bahar Bandani - Susan, (2021) | Derya Çınar (2021) | Elaheh Ghanbari(2021) | Ezgi Bilmiç  (2023) | Franziska Springer  (2024) | Haiyan Hao  (2022) | Hatice BALCI  (2024) | Hiromi Okuyama(2024) | I - Ching Hou (2020) | Jolien M.Admiraal(2017) | Julia D.H.P.Si(2024) | Kaina Zhou (2020) |
| **Bias arising from the randomization process** | 1.1 Was the allocation sequence random? | Y | Y | Y | Y | Y | Y | Y | Y | Y | Y | Y | Y |
|  | 1.2 Was the allocation sequence concealed until participants were enrolled and assigned to interventions? | PY | PY | Y | Y | PY | PY | Y | PY | PY | Y | Y | Y |
|  | 1.3 Did baseline differences between intervention groups suggest a problem with the randomization process? | N | N | N | N | N | N | N | N | N | N | N | N |
|  | **Risk of bias judgement** | Low | Low | Low | Low | Low | Low | Low | Low | Low | Low | Low | Low |
| **Bias due to deviations from intended interventions** | 2.1.Were participants aware of their assigned intervention during the trial? | Y | Y | Y | Y | Y | Y | Y | Y | Y | Y | Y | Y |
|  | 2.2.Were carers and people delivering the interventions aware of participants' assigned intervention during the trial? | Y | Y | Y | Y | Y | Y | Y | Y | Y | Y | Y | Y |
|  | 2.3. If Y/PY/NI to 2.1 or 2.2: Were there deviations from the intended intervention that arose because of the experimental context? | N | N | PN | N | N | N | N | N | N | PN | PY | PN |
|  | 2.4 If Y/PY to 2.3: Were these deviations likely to have affected the outcome? | NA | NA | NA | NA | NA | NA | NA | NA | NA | NA | PN | NA |
|  | 2.5. If Y/PY/NI to 2.4: Were these deviations from intended intervention balanced between groups? | NA | NA | NA | NA | NA | NA | NA | NA | NA | NA | NA | NA |
|  | 2.6 Was an appropriate analysis used to estimate the effect of assignment to intervention? | Y | Y | Y | Y | Y | Y | Y | Y | Y | Y | Y | Y |
|  | 2.7 If N/PN/NI to 2.6: Was there potential for a substantial impact (on the result) of the failure to analyse participants in the group to which they were randomized? | NA | NA | NA | NA | NA | NA | NA | NA | NA | NA | NA | NA |
|  | **Risk of bias judgement** | Low | Low | Low | Low | Low | Low | Low | Low | Low | Low | Some concerns | Low |
| **Bias due to missing outcome data** | 3.1 Were data for this outcome available for all, or nearly all, participants randomized? | N | Y | Y | Y | Y | Y | Y | Y | Y | N | PY | Y |
|  | 3.2 If N/PN/NI to 3.1: Is there evidence that result was not biased by missing outcome data? | PN | NA | NA | NA | NA | NA | NA | NA | NA | PY | NA | NA |
|  | 3.3 If N/PN to 3.2: Could missingness in the outcome depend on its true value? | PY | NA | NA | NA | NA | NA | NA | NA | NA | NA | NA | NA |
|  | 3.4 If Y/PY/NI to 3.3: Is it likely that missingness in the outcome depended on its true value? | PY | NA | NA | NA | NA | NA | NA | NA | NA | NA | NA | NA |
|  | **Risk of bias judgement** | High | Low | Low | Low | Low | Low | Low | Low | Low | Low | Low | Low |
| **Bias in measurement of the outcome** | 4.1 Was the method of measuring the outcome inappropriate? | N | N | N | N | N | N | N | N | N | N | N | N |
|  | 4.2 Could measurement or ascertainment of the outcome have differed between intervention groups? | N | N | N | N | N | N | N | N | N | N | N | PN |
|  | 4.3 Were outcome assessors aware of the intervention received by study participants? | Y | Y | PY | N | N | N | N | N | N | PN | PN | N |
|  | 4.4 If Y/PY/NI to 4.3: Could assessment of the outcome have been influenced by knowledge of intervention received? | PY | N | PY | NA | NA | NA | NA | NA | NA | NA | NA | NA |
|  | 4.5 If Y/PY/NI to 4.4: Is it likely that assessment of the outcome was influenced by knowledge of intervention received? | PY | NA | PY | NA | NA | NA | NA | NA | NA | NA | NA | NA |
|  | **Risk of bias judgement** | High | Low | High | Low | Low | Low | Low | Low | Low | Low | Low | Low |
| **Bias in selection of the reported result** | 5.1 Were the data that produced this result analysed in accordance with a pre-specified analysis plan that was finalized before unblinded outcome data were available for analysis? | Y | Y | Y | Y | Y | Y | Y | Y | Y | Y | Y | Y |
|  | 5.2 ... multiple eligible outcome measurements (e.g. scales, definitions, time points) within the outcome domain? | N | N | N | N | N | N | N | N | N | N | N | N |
|  | 5.3 ... multiple eligible analyses of the data? | N | N | N | N | N | N | N | N | N | N | N | N |
|  | **Risk of bias judgement** | Low | Low | Low | Low | Low | Low | Low | Low | Low | Low | Low | Low |
| **Overall bias** | **Risk of bias judgement** | High | Low | High | Low | Low | Low | Low | Low | Low | Low | Some concerns | Low |

***Notes: ‘Y’ indicates yes, ‘PY’ indicates probably yes, ‘PN’ indicates probably no, ‘N’ indicates no, ‘NI’ indicates no information, ‘NA’ indicates not applicable.***

# Table S2-3. Details of risk of bias assessment of included RCTs

| **Domain** | **Signaling question** | **Response** | | | | | | | | | | | |
| --- | --- | --- | --- | --- | --- | --- | --- | --- | --- | --- | --- | --- | --- |
|  |  | Simon Sebastia(2020) | Sue V.Petzel(2018) | Tamara Somers  (2016) | Anemiek Visser  (2018) | Xi Chen  (2024) | Xiaosheng Dong  (2019) | Yanfei Xu  (2021) | Yiling Sui  (2020) | Ying Wang  (2021) | Yuan Yu  (2022) | Zhiyou Peng  (2020) | Jiemin Zhu  (2018) |
| **Bias arising from the randomization process** | 1.1 Was the allocation sequence random? | Y | Y | Y | Y | Y | Y | Y | Y | Y | Y | Y | Y |
|  | 1.2 Was the allocation sequence concealed until participants were enrolled and assigned to interventions? | PY | Y | PY | PN | Y | Y | PY | PY | Y | Y | Y | Y |
|  | 1.3 Did baseline differences between intervention groups suggest a problem with the randomization process? | N | N | N | N | N | N | N | N | N | N | N | N |
|  | **Risk of bias judgement** | Low | Low | Low | High | Low | Low | Low | Low | Low | Low | Low | Low |
| **Bias due to deviations from intended interventions** | 2.1.Were participants aware of their assigned intervention during the trial? | Y | PY | Y | Y | Y | Y | Y | Y | Y | Y | Y | Y |
|  | 2.2.Were carers and people delivering the interventions aware of participants' assigned intervention during the trial? | Y | PY | Y | Y | Y | Y | Y | Y | Y | Y | Y | Y |
|  | 2.3. If Y/PY/NI to 2.1 or 2.2: Were there deviations from the intended intervention that arose because of the experimental context? | PN | PN | PN | N | N | N | N | PN | PN | PN | N | PN |
|  | 2.4 If Y/PY to 2.3: Were these deviations likely to have affected the outcome? | NA | NA | NA | NA | NA | NA | NA | NA | NA | NA | NA | NA |
|  | 2.5. If Y/PY/NI to 2.4: Were these deviations from intended intervention balanced between groups? | NA | NA | NA | NA | NA | NA | NA | NA | NA | NA | NA | NA |
|  | 2.6 Was an appropriate analysis used to estimate the effect of assignment to intervention? | Y | Y | Y | Y | Y | Y | Y | Y | Y | Y | Y | Y |
|  | 2.7 If N/PN/NI to 2.6: Was there potential for a substantial impact (on the result) of the failure to analyse participants in the group to which they were randomized? | NA | NA | NA | NA | NA | NA | NA | NA | NA | NA | NA | NA |
|  | **Risk of bias judgement** | Low | Low | Low | Low | Low | Low | Low | Low | Low | Low | Low | Low |
| **Bias due to missing outcome data** | 3.1 Were data for this outcome available for all, or nearly all, participants randomized? | PY | PY | N | PY | Y | PY | Y | PY | PY | Y | Y | Y |
|  | 3.2 If N/PN/NI to 3.1: Is there evidence that result was not biased by missing outcome data? | NA | NA | PN | NA | NA | NA | NA | NA | NA | NA | NA | NA |
|  | 3.3 If N/PN to 3.2: Could missingness in the outcome depend on its true value? | NA | NA | PY | NA | NA | NA | NA | NA | NA | NA | NA | NA |
|  | 3.4 If Y/PY/NI to 3.3: Is it likely that missingness in the outcome depended on its true value? | NA | NA | PY | NA | NA | NA | NA | NA | NA | NA | NA | NA |
|  | **Risk of bias judgement** | Low | Some concerns | High | Low | Low | Low | Low | Low | Some concerns | Low | Low | Low |
| **Bias in measurement of the outcome** | 4.1 Was the method of measuring the outcome inappropriate? | N | N | N | N | N | N | N | N | N | N | N | N |
|  | 4.2 Could measurement or ascertainment of the outcome have differed between intervention groups? | N | PN | N | N | N | N | PN | PN | N | N | N | N |
|  | 4.3 Were outcome assessors aware of the intervention received by study participants? | N | PN | N | N | N | PN | PN | PN | N | N | PN | N |
|  | 4.4 If Y/PY/NI to 4.3: Could assessment of the outcome have been influenced by knowledge of intervention received? | NA | NA | NA | NA | NA | NA | NA | NA | NA | NA | NA | NA |
|  | 4.5 If Y/PY/NI to 4.4: Is it likely that assessment of the outcome was influenced by knowledge of intervention received? | NA | NA | NA | NA | NA | NA | NA | NA | NA | NA | NA | NA |
|  | **Risk of bias judgement** | Low | Low | Low | Low | Low | Low | Low | Low | Low | Low | Low | Low |
| **Bias in selection of the reported result** | 5.1 Were the data that produced this result analysed in accordance with a pre-specified analysis plan that was finalized before unblinded outcome data were available for analysis? | Y | Y | Y | Y | Y | Y | Y | Y | PY | PY | Y | PY |
|  | 5.2 ... multiple eligible outcome measurements (e.g. scales, definitions, time points) within the outcome domain? | N | N | N | N | N | N | N | N | N | N | N | N |
|  | 5.3 ... multiple eligible analyses of the data? | N | N | N | N | N | N | N | N | N | N | N | N |
|  | **Risk of bias judgement** | Low | Low | Low | Low | Low | Low | Low | Low | Low | Low | Low | Low |
| **Overall bias** | **Risk of bias judgement** | Low | Some concerns | High | High | Low | Low | Low | Low | Some concerns | Low | Low | Low |

***Notes: ‘Y’ indicates yes, ‘PY’ indicates probably yes, ‘PN’ indicates probably no, ‘N’ indicates no, ‘NI’ indicates no information, ‘NA’ indicates not applicable.***

# Table S3: Subgroup Analysis- Fatigue

| **Subgroup categories** | **Subgroup name** | **Number of Studies** | **Pooled Effect Size SMD (95% CI)** | **Heterogeneity (I²)** |
| --- | --- | --- | --- | --- |
| Region | Asia | 6 | -1.00 [-2.05, 0.06] | 97% |
|  | Europe | 4 | -0.10 [-0.23, 0.04] | 40% |
| App | WeChat | 3 | -0.50 [-1.72, 0.72] | 96% |
|  | Non - WeChatt | 9 | -0.54 [-1.02, -0.05] | 96% |
| Intervention Time | <3months | 2 | -0.01 [-0.34, 0.31] | 0% |
|  | >3months | 10 | -0.61 [-1.10, -0.12] | 96% |

# Table S4: Subgroup Analysis- Anxiety

| **Subgroup categories** | **Subgroup name** | **Number of Studies** | **Pooled Effect Size SMD (95% CI)** | **Heterogeneity (I²)** |
| --- | --- | --- | --- | --- |
| Region | Asia | 10 | -0.95 [-1.31, -0.60] | 88% |
|  | America | 2 | 0.15 [-0.43, 0.73] | 54% |
|  | Europe | 3 | -0.10 [-0.25, 0.04] | 0 |
| App | WeChat | 7 | -1.08 [-1.51, -0.64] | 90% |
|  | Non - WeChatt | 9 | -0.21 [-0.44, 0.03] | 74% |
| Intervention Time | <3months | 9 | -0.52 [-0.89, -0.16] | 87% |
|  | >3months | 7 | -0.71 [-1.22, -0.20] | 95% |

# Table S5: Subgroup Analysis- Depression

| **Subgroup categories** | **Subgroup name** | **Number of Studies** | **Pooled Effect Size SMD (95% CI)** | **Heterogeneity (I²)** |
| --- | --- | --- | --- | --- |
| Region | Asia | 7 | -0.58 [-0.90, -0.26] | 83% |
|  | Europe | 3 | -0.23 [-0.38, -0.09] | 0 |
| App | WeChat | 6 | -0.68 [-1.00, -0.37] | 80% |
|  | Non - WeChatt | 6 | -0.07 [-0.27, 0.14] | 55% |
| Intervention Time | <3months | 6 | -0.35 [-0.61, -0.10] | 76% |
|  | >3months | 6 | -0.34 [-0.77, 0.08] | 86% |

# Table S6: Subgroup Analysis- Quality of Life

| **Subgroup categories** | **Subgroup name** | **Number of Studies** | **Pooled Effect Size SMD (95% CI)** | **Heterogeneity (I²)** |
| --- | --- | --- | --- | --- |
| Region | Asia | 16 | 0.69 [0.39, 0.99] | 90% |
|  | Europe | 7 | 0.22 [-0.08, 0.52] | 81% |
| App | WeChat | 8 | 0.78 [0.32, 1.25] | 92% |
|  | Non - WeChatt | 19 | 0.56 [0.28, 0.83] | 91% |
| Intervention Time | <3months | 5 | 1.10 [0.59, 1.62] | 87% |
|  | >3months | 22 | 0.52 [0.26, 0.78] | 92% |

# Table S7 : Sensitivity analysis (leave-one-out method) - Fatigue

| **Research removal** | **The pooled effect size SMD (95% CI) after removing this research** | **Heterogeneity (I²)** |
| --- | --- | --- |
| Bahar Bandani - Susan, 2021 | -0.55 [-1.01, -0.10] | 96% |
| Ezgi Bilmiç, 2023 | -0.57 [-1.04, -0.11] | 96% |
| T1 Franziska Springer, 2024 | -0.58 [-1.05, -0.11] | 96% |
| T2 Franziska Springer, 2024 | -0.58 [-1.04, -0.12] | 96% |
| Kaina Zhou, 2020 | -0.60 [-1.05, -0.14] | 96% |
| Limin Xia, 2020 | -0.28 [-0.57, 0.01] | 89% |
| Liping Wang, 2024 | -0.59 [-1.05, -0.13] | 96% |
| N. Kearney, 2009 | -0.55 [-1.01, -0.08] | 96% |
| Roy A. Willems, 2017 | -0.56 [-1.06, -0.05] | 96% |
| Simon Sebastian Spahrkäs, 2020 | -0.55 [-1.06, -0.05] | 96% |
| Ying Wang, 2021 | -0.39 [-0.81, 0.03] | 95% |
| Yuan Yu, 2022 | -0.46 [-0.90, -0.02] | 95% |

Notes: T1, Trial 1; T2, Trial 2.

# Table S8 : Sensitivity analysis (leave-one-out method) - Anxiety

| **Research removal** | **The pooled effect size SMD (95% CI) after removing this research** | **Heterogeneity (I²)** |
| --- | --- | --- |
| Elaheh Ghanbari, 2021 | -0.57 [-0.88, -0.26] | 92% |
| Ezgi Bilmiç, 2023 | -0.65 [-0.96, -0.34] | 91% |
| T1 Franziska Springer, 2024 | -0.64 [-0.96, -0.32] | 92% |
| T2 Franziska Springer, 2024 | -0.63 [-0.95, -0.31] | 92% |
| Jiemin Zhu, 2018 | -0.63 [-0.95, -0.32] | 92% |
| Limin Xia, 2020 | -0.53 [-0.81, -0.25] | 89% |
| T1 Meihua Zheng, 2022 | -0.61 [-0.93, -0.29] | 92% |
| T2 Meihua Zheng, 2022 | -0.61 [-0.92, -0.29] | 92% |
| Mihir Kamdar, 2024 | -0.67 [-0.97, -0.37] | 91% |
| Pardis Doosti, 2024 | -0.59 [-0.90, -0.28] | 92% |
| Roy A. Willems, 2017 | -0.64 [-0.96, -0.32] | 91% |
| Sue V.Petzel, 2018 | -0.62 [-0.93, -0.31] | 92% |
| Xi Chen, 2024 | -0.57 [-0.88, -0.26] | 92% |
| Yiling Sui, 2020 | -0.61 [-0.94, -0.29] | 91% |
| Ying Wang, 2021 | -0.48 [-0.75, -0.22] | 89% |
| Zhiyou Peng, 2020 | -0.58 [-0.89, -0.26] | 91% |

Notes: T1, Trial 1; T2, Trial 2.

# Table S9 : Sensitivity analysis (leave-one-out method) - Depression

| **Research removal** | **The pooled effect size SMD (95% CI) after removing this research** | **Heterogeneity (I²)** |
| --- | --- | --- |
| Ezgi Bilmiç, 2023 | -0.42 [-0.64, -0.20] | 79% |
| T1 Franziska Springer, 2024 | -0.37 [-0.62, -0.12] | 83% |
| T2 Franziska Springer, 2024 | -0.36 [-0.60, -0.12] | 83% |
| Jiemin Zhu, 2018 | -0.40 [-0.63, -0.17] | 81% |
| T1 Meihua Zheng, 2022 | -0.35 [-0.59, -0.11] | 83% |
| T2 Meihua Zheng, 2022 | -0.36 [-0.60, -0.12] | 83% |
| Roy A. Willems, 2017 | -0.40 [-0.62, -0.18] | 81% |
| Xi Chen, 2024 | -0.28 [-0.47, -0.09] | 72% |
| Yiling Sui, 2020 | -0.35 [-0.60, -0.11] | 83% |
| Ying Wang, 2021 | -0.36 [-0.58, -0.14] | 81% |
| Zhiyou Peng, 2020 | -0.35 [-0.60, -0.10] | 83% |

Notes: T1, Trial 1; T2, Trial 2.

# Table S10 : Sensitivity analysis (leave-one-out method) - Quality of life

| **Research removal** | **The pooled effect size SMD (95% CI) after removing this research** | **Heterogeneity (I²)** |
| --- | --- | --- |
| Annemiek Visser, 2018 | 0.65 [0.40, 0.90] | 92% |
| Derya Çınar, 2021 | 0.57 [0.34, 0.81] | 91% |
| T1 Franziska Springer, 2024 | 0.66 [0.41, 0.90] | 92% |
| T2 Franziska Springer, 2024 | 0.66 [0.42, 0.91] | 92% |
| Haiyan Hao, 2022 | 0.58 [0.34, 0.81] | 92% |
| Hatice BALCI, 2024 | 0.57 [0.34, 0.81] | 91% |
| Hiromi Okuyama, 2024 | 0.65 [0.40, 0.90] | 92% |
| I-Ching Hou, 2020 | 0.65 [0.41, 0.90] | 92% |
| Jiemin Zhu, 2018 | 0.63 [0.38, 0.88] | 92% |
| Jolien M.Admiraal, 2017 | 0.65 [0.40, 0.90] | 92% |
| Kaina Zhou, 2020 | 0.63 [0.38, 0.88] | 92% |
| Lauren J. Frensham, 2018 | 0.65 [0.40, 0.89] | 92% |
| Limin Xia, 2020 | 0.64 [0.39, 0.89] | 92% |
| Liping Wang, 2024 | 0.61 [0.36, 0.85] | 92% |
| Noelia Galiano-Castillo, 2016 | 0.61 [0.36, 0.85] | 92% |
| Pardis Doosti, 2024 | 0.62 [0.38, 0.87] | 92% |
| Patricia Martínez - Miranda, 2024 | 0.61 [0.36, 0.85] | 92% |
| Peng Zhou, 2024 | 0.58 [0.35, 0.81] | 91% |
| Roy A. Willems, 2017 | 0.65 [0.40, 0.91] | 92% |
| Simon Sebastian Spahrkäs, 2020 | 0.65 [0.40, 0.91] | 92% |
| Xiaosheng Dong, 2019 | 0.63 [0.38, 0.88] | 92% |
| Xi Chen, 2024 | 0.60 [0.36, 0.85] | 92% |
| Yanfei Xu, 2021 | 0.59 [0.36, 0.83] | 92% |
| Yiling Sui, 2020 | 0.64 [0.39, 0.89] | 92% |
| Ying Wang, 2021 | 0.68 [0.44, 0.91] | 92% |
| Yuan Yu, 2022 | 0.61 [0.36, 0.85] | 92% |
| Zhiyou Peng, 2020 | 0.63 [0.38, 0.89] | 92% |

Notes: T1, Trial 1; T2, Trial 2.

# Table S11. GRADE assessments for the certainty of evidence of outcomes.

Question: Remote Online Intervention compared to Usual care for Cancer Patients

| **Certainty assessment** | | | | | | | **№ of patients** | | **Effect** | | **Certainty** | **Importance** |
| --- | --- | --- | --- | --- | --- | --- | --- | --- | --- | --- | --- | --- |
| **№ of studies** | **Study design** | **Risk of bias** | **Inconsistency** | **Indirectness** | **Imprecision** | **Other considerations** | **Remote Online Intervention** | **Usual care** | **Relative (95% CI)** | **Absolute (95% CI)** |  |  |
| **Anxiety** | | | | | | | | | | | | |
| 14 | randomised trials | not serious | very serious^a^ | not serious | not serious | none | 1109 | 1172 | - | SMD **0.6 lower** (0.9 lower to 0.3 lower) | ⨁⨁◯◯ Low^a^ |  |
| **Depression** | | | | | | | | | | | | |
| 10 | randomised trials | not serious | very serious^b^ | not serious | not serious | none | 912 | 981 | - | SMD **0.36 lower** (0.58 lower to 0.14 lower) | ⨁⨁◯◯ Low^b^ |  |
| **Quality of life** | | | | | | | | | | | | |
| 26 | randomised trials | not serious | very serious^c^ | not serious | not serious | none | 1858 | 1917 | - | SMD **0.63 higher** (0.39 higher to 0.87 higher) | ⨁⨁◯◯ Low^c^ |  |
| **Fatigue** | | | | | | | | | | | | |
| 11 | randomised trials | not serious | very serious^d^ | not serious | not serious | none | 1002 | 1057 | - | SMD **0.52 lower** (0.95 lower to 0.09 lower) | ⨁⨁◯◯ Low^d^ |  |
| **Pain** | | | | | | | | | | | | |
| 14 | randomised trials | not serious | very serious^e^ | not serious | not serious | none | 785 | 774 | - | SMD **0.33 lower** (0.58 lower to 0.09 lower) | ⨁⨁◯◯ Low^e^ |  |

CI: confidence interval; SMD: standardised mean difference

#### Explanations

a. Very serious inconsistency, downgraded two levels due to high heterogeneity (I² = 91%).

b. Very serious inconsistency, downgraded two levels due to high heterogeneity (I² = 81%).

c. Very serious inconsistency, downgraded two levels due to high heterogeneity (I² = 92%).

d. Very serious inconsistency, downgraded two levels due to high heterogeneity (I² = 95%).

e. Very serious inconsistency, downgraded two levels due to high heterogeneity (I² =82%).
