# Supplementary material for: Effects of Remote Web-Based Interventions on the Physiological and Psychological States of Patients With Cancer: Systematic Review With Meta-Analysis
Source: JMIR Mhealth Uhealth. 2025 Jun 12;13:e71196. doi: 10.2196/71196 (PMC12203031; doi:10.2196/71196)
Supplement: Multimedia Appendix 3 [file mhealth_v13i1e71196_app3.docx]

**Multimedia Appendix 3** Characteristics of the included studies (randomized controlled trials) (continued from Table 1).

| Author, year | Participants | | | |
| --- | --- | --- | --- | --- |
|  | Sample size | Age  (Range or intervention group / control group) | Sex (Male / Female) | Types of Cancer |
| Yiling Sui, 2020 | 200 | 61.37 ± 11.21 / 62.35 ± 9.98 | 164 / 36 | Non-small cell lung cancer |
| Zhiyou Peng, 2020 | 302 | 55.6±6.8 / 56.3±7.0 | 144/158 | Not specified |
| Bahar Bandani - Susan, 2021 | 38 | 46.79 ± 12.28/45.89 ± 7.64 | 0/38 | Breast cancer |
| Yanfei Xu, 2021 | 126 | 48.74±6.88 / 47.13±7.58 | 0/126 | Breast cancer |
| Haiyan Hao, 2022 | 80 | 64.8±12.58 / 63.25±17.16 | Not specified | Urinary system cancers (prostate, testicular, penile, kidney, ureter, bladder) |
| Qiuling Zhao, 2024 | 42 | 51-75 | 23/19 | Multiple cancers |
| Ying Wang, 2021 | 72 | ≥18 | 40/32 | Acute leukaemia |
| Li - Ping Wang, 2024 | 125 | 40.29±19.73/40.53±22.00 | 0/125 | Breast cancer |
| Meihua Zheng, 2022 | 150 | Trial1: 57.48 ±9.29/58.46 ±9.96;Trial 2: 59.50 ±10.67/58.46 ±9.96 | 123/27 | Digestive system cancers (colon, rectum, stomach, liver, esophagus) |
| Yuan Yu, 2022 | 168 | ≥18 | 139/29 | Esophageal cancer |
| Tamara J. Somers, 2016 | 30 | 60±11 | 15/15 | Cancers (breast, prostate, lung, colorectal) |
| Mihir Kamdar, 2024 | 112 | 54.6±10.9/50.7±12.4 | 57/55 | Multiple cancers |
| Ezgi Bilmiç, 2023 | 110 | ≥18 | 37/73 | Multiple cancers |
| Patricia Martínez - Miranda, 2024 | 49 | 49.21±5.91/50.00±8.04 | 0/49 | Breast cancer |
| Julia D.H.P. Simon, 2024 | 158 | 7.5±5.1/7.5±5.4 | 79/79 | Hematology - oncology, neuro - oncology, and solid tumors |
| Hiromi Okuyama, 2024 | 125 | 63.9±7.8/62.7±7.4 | 1/124 | Breast cancer |
| N. Kearney, 2009 | 112 | 55.1±10.6/56.9±10.5 | 26/86 | Cancers (breast, lung, colorectal cancer) |
| Annemiek Visser, 2018 | 109 | 55.8 ± 8.3 / 57.9 ± 8.8 | 0/109 | Breast cancer |
| Jiemin Zhu, 2018 | 114 | 46.2±8.5/47.2±8.3 | 0/114 | Breast cancer |
| Kaina Zhou, 2020 | 111 | 49.84±8.85/49.98±9.84 | 0/111 | Breast cancer |
| Limin Xia, 2020 | 155 | 18-70 | 83/72 | Low rectal cancer |
| Xi Chen, 2024 | 91 | 48.09±8.25/50.59 ±6.10 | 0/91 | Breast cancer |
| Hatice BALCI, 2024 | 81 | 47.04±10.21/54.24±12.92 | 0/81 | Breast cancer |
| Derya Çınar, 2021 | 64 | 45.9 ± 8.3/45.5 ± 9.8 | 0/64 | Breast cancer |
| Roy A. Willems, 2017 | 409 | 56.26±10.84/56.28±11.45 | 77/332 | Multiple cancers |
| Elaheh Ghanbari, 2021 | 82 | 46.9±9.83/46±8.80 | 0 / 82 | Breast cancer |
| Peng Zhou, 2024 | 120 | 62.72 ± 7.91/61.78 ± 11.80 | 79/41 | Rectal cancer |
| Noelia Galiano-Castillo, 2016 | 76 | 47.4 ± 9.6/49.2 ± 7.9 | 0/76 | Breast cancer |
| I - Ching Hou, 2020 | 112 | ＞20 | 0/112 | Breast cancer |
| Jolien M.Admiraal, 2017 | 125 | ＞18 | 0/125 | Breast cancer |
| Xiaosheng Dong, 2019 | 50 | 48±5.54/51.63±7.49 | 0/50 | Breast cancer |
| Lauren J. Frensham, 2018 | 91 | 65.2±9.3/66.1±9.4 | 44/47 | Multiple cancers |
| Sue V. Petzel, 2018 | 29 | 59.6±10.0/55.5±8.4 | 0/29 | Ovarian cancer |
| Pardis Doosti, 2024 | 68 | 49.41±8.80 / 50.26±13.64 | 0/68 | Cancers (ovarian, cervical, uterus, vaginal) |
| Franziska Springer, 2024 | 218 | 55±11/58±10 | 132/86 | Multiple cancers |
| Simon Sebastian Spahrkäs, 2020 | 417 | 56.7 ± 9.99/56.2 ± 9.42 | N/A | Multiple cancers |
